# Enhancing Cycling Stability of Aqueous Aluminum‐Metal Batteries via LaCl_3_‐Modulated Interfacial Reactions

**DOI:** 10.1002/advs.202514322

**Published:** 2025-12-19

**Authors:** Yanshen Gao, Karol Załęski, Emerson Coy, Błażej Scheibe, Kacper Szymański, Qingshan Yang, Ewa Mijowska, Xianjie Liu, Ran Jia, Dariusz Moszyński, Linfeng Hu, Rudolf Holze, Xuecheng Chen

**Affiliations:** ^1^ Faculty of Chemical Technology and Engineering West Pomeranian University of Technology in Szczecin Piastów Ave. 42 Szczecin 71‐065 Poland; ^2^ NanoBioMedical Centre Adam Mickiewicz University Wszechnicy Piastowskiej 3 Poznan 61–614 Poland; ^3^ Center for Advanced Materials and Manufacturing Process Engineering (CAMMPE) West Pomeranian University of Technology in Szczecin Szczecin 71‐065 Poland; ^4^ Department of Science and Technology Laboratory of Organic Electronics (LOE) Linköping University Norrköping 60174 Sweden; ^5^ State Key Laboratory of Inorganic Synthesis and Preparative Chemistry College of Chemistry Jilin University Changchun Jilin 130012 China; ^6^ School of Materials Science and Engineering Southeast University Nanjing 211189 China; ^7^ Chemnitz University of Technology D‐09107 Chemnitz Germany; ^8^ State Key Laboratory of Materials‐oriented Chemical Engineering School of Energy Science and Engineering Nanjing Tech University Nanjing Jiangsu 211816 China

**Keywords:** AlCl_3_, aluminum metal anode, aqueous aluminum‐metal batteries, LaCl_3_

## Abstract

Aluminum metal is considered an ideal candidate for aqueous metal batteries due to its abundant availability and high theoretical capacity (2980 mAh g^−1^). However, the development of aqueous aluminum‐metal batteries is significantly hindered by the detrimental side reactions (such as solvent decomposition, Al corrosion, and passivation) that occur when aluminum metal is in contact with aqueous electrolytes. In this paper, introducing trace amounts of lanthanum chloride (LaCl_3_) into the low‐cost yet highly corrosive aqueous aluminum chloride (AlCl_3_) solution as an electrolyte for aqueous aluminum‐metal batteries is proposed. The additional halide ions introduced into the electrolyte system modulate the solvation structure of Al^3+^, while the electrochemically inert La^3+^ induces a transformation of the aluminum metal interface from aggressive, localized penetration corrosion to more controlled, uniform corrosion, thereby enabling long‐lasting and stable electrochemical reactions. In a full battery test using Prussian blue analogs (PBA) as the cathode material and aluminum metal as the anode material, the average Coulombic efficiency exceeded 97%, and the cycling stability is 74.4% at a current density of 250 mA g^−1^ over 800 cycles.

## Graphical abstract

In the aqueous AlCl_3_ electrolyte of aluminum‐metal batteries, the introduction of LaCl_3_ adjusts the solvation structure of Al^3+^ and enhances its diffusion level. The oxide precipitate formed by La^3+^ covers the aluminum metal anode and effectively alleviates corrosion reactions, thereby improving the cycling stability of the battery.

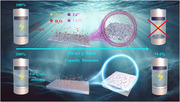

## 1 Introduction

**Figure 1:**
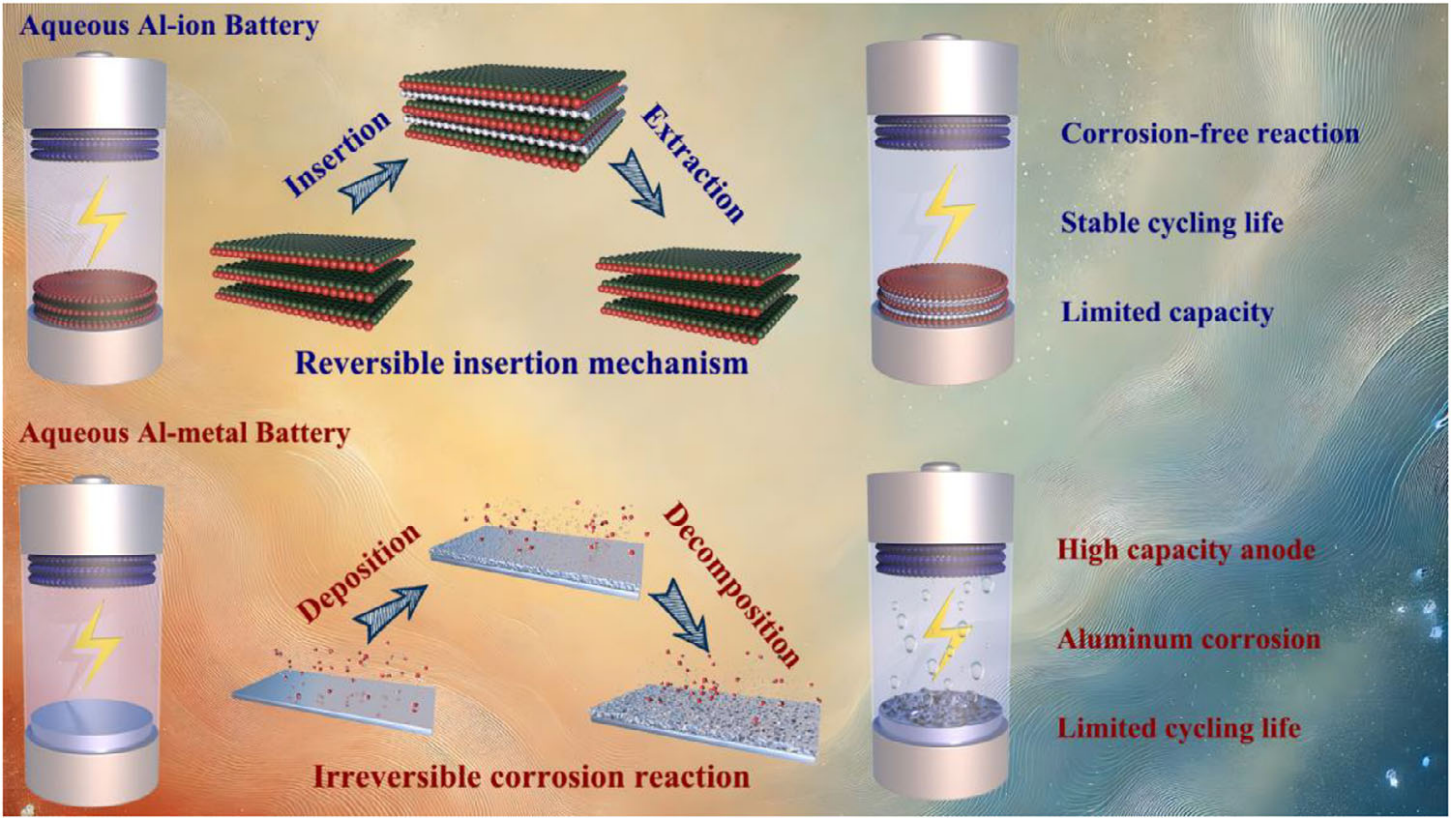
Reaction mechanisms, advantages, and disadvantages of different types of Al‐ion batteries.

Despite the tremendous success of lithium‐ion batteries in the energy storage field, the high cost and low safety of lithium metal, along with the negative reactions of organic electrolytes, have limited their further development.^[^ ^1^, ^2^, ^3^ ^]^ Due to the high abundance of aluminum in the Earth's crust, its environmental friendliness, low cost, and high gravimetric and volumetric capacities, the use of aluminum as an anode material and its application in battery energy storage systems have become the focus of current research in the energy field.^[^ ^4^, ^5^, ^6^, ^7^ ^]^ Based on the electrolyte systems, research on aluminum‐ion batteries can be broadly classified into two types: non‐aqueous electrolytes with ionic liquid systems and aqueous electrolytes with water as the solvent. Compared to nonaqueous ionic liquid electrolytes, aqueous electrolytes have the advantages of low cost, high conductivity, and high safety.^[^ ^8^, ^9^ ^]^ At the current stage, aqueous aluminum‐ion batteries can be classified into two categories (**Figure** **1**): aluminum‐ion batteries without aluminum metal as the anode and aluminum‐ion batteries (or aluminum‐metal batteries) that use aluminum metal as the anode. Since no aluminum metal is involved in the reaction, the interior of an aluminum‐ion battery without an Al anode can usually remain stable for long periods during cycling. However, their actual capacity is strictly constrained by the properties of the cathode and anode materials. In aqueous aluminum‐metal batteries, although the aluminum metal anode possesses a high theoretical capacity, the severe corrosion induced by the aggressive electrolyte accelerates the depletion of the aqueous solvent and triggers side reactions, such as gas evolution (particularly in AlCl_3_‐based electrolytes), ultimately leading to a severely limited cycling lifespan.^[^ ^10^, ^11^, ^12^ ^]^ To address this issue, recent research on aqueous aluminum‐ion batteries has focused on using aluminum trifluoromethanesulfonate (Al(OTF)_3_) as a chemically milder electrolyte, gradually replacing the highly reactive AlCl_3_ electrolyte. Based on this, a series of modification methods, such as aluminum‐metal interfacial engineering,^[^ ^13^, ^14^ ^]^ electrolyte modification,^[^ ^15^, ^16^, ^17^ ^]^ salt‐in‐water strategies^[^ ^18^, ^19^ ^]^ and aluminum metal anode alloying,^[^ ^20^, ^21^ ^]^ have been adopted to prolong the cycling life. From a cost perspective, however, compared to the expensive Al(OTF)_3_, the cheaper AlCl_3_ still holds research potential as an electrolyte for aqueous aluminum‐metal batteries, especially when high‐concentration electrolytes are used, where the electrolyte cost becomes a crucial factor. However, the issues of high corrosion of aluminum metal by AlCl_3_ electrolyte in aqueous environments and gas release due to corrosion remain serious obstacles to its large‐scale application.

Recent advances in corrosion science have demonstrated that the addition of rare earth elements or additives greatly enhances the protective properties of alloys and offers promising solutions for dealing with aggressive marine environments.^[^ ^22^, ^23^, ^24^, ^25^ ^]^ From the perspective of aluminum metal corrosion, similar to seawater corrosion, the corrosion of the aluminum metal interface by Cl^−^ in the AlCl_3_ electrolyte is a major cause of the dramatic decrease in cycle life of aqueous aluminum‐metal batteries. Motivated by this discovery, a trace amount of LaCl_3_ was added to the aqueous AlCl_3_ electrolyte. Studies have shown that La^3+^ in the electrolyte is enriched in the corrosion‐prone sites on the surface of aluminum metal and achieves precipitation coverage, which effectively mitigates the corrosion and penetration of the acidic electrolyte into the anode of aluminum metal, and contributes to the transformation of the corrosion reaction at the interface from deep and localized corrosion to homogeneous corrosion. Additionally, the halogen anion (Cl^−^) introduced into the electrolyte reduces the coordination effect between aluminum ions and water molecules. The aluminum‐metal battery assembled with PBA as the cathode material and aluminum metal as the anode material was stably cycled for 800 cycles, achieving a capacity retention of 74.4% and an average Coulombic efficiency of more than 97% at a current density of 250 mA g^−1^ with 1 m AlCl_3_ as the electrolyte. The remarkable cycling stability is mainly attributed to the effective regulation of the interfacial reaction of La^3+^ on aluminum metal.

## 2 Results and Discussion

### 2.1 Preparation and Physical Characterization of PBA and Aqueous Electrolytes

**Figure 2:**
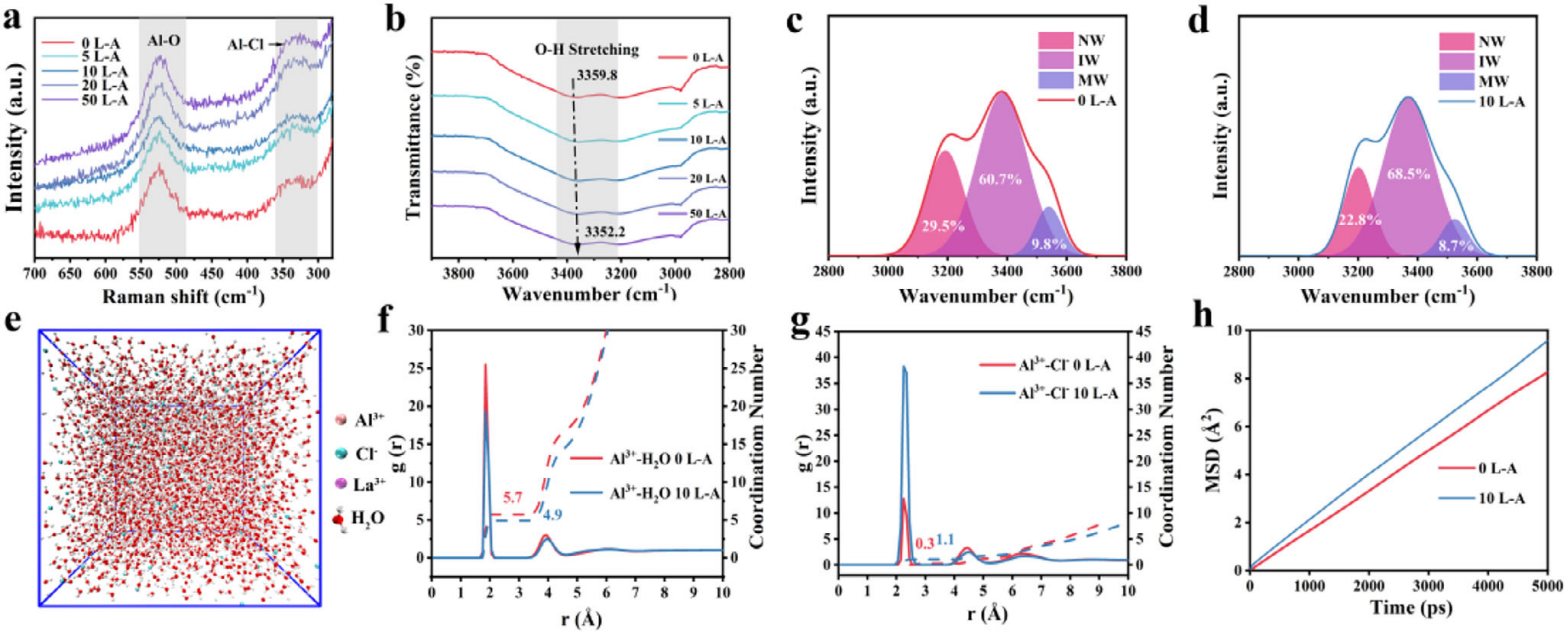
Characterization and simulation of LaCl_3_‐regulated solvation structures. a) Raman spectra of different electrolytes. b) FT‐IR spectra of different electrolytes. c) and d) Fitted curves of FT‐IR spectra. e) The snapshot of MD simulation trajectories of 10 L‐A. f) Radial distribution functions and coordination numbers of Al‐H_2_O in 0 L‐A and 10 L‐A electrolytes. g) Radial distribution functions and coordination numbers of Al‐Cl^−^ in 0 L‐A and 10 L‐A electrolytes. h) MSD curve in 0 L‐A and 10 L‐A electrolytes systems from MD simulations.

The X‐ray diffraction (XRD) patterns and scanning electron microscopy (SEM) images of cobalt hexacyanoferrate (CoHCF) Prussian blue analog cathode materials synthesized via the co‐precipitation method are shown in Figures S1 and S2 (Supporting Information). The crystal structure of CoHCF adopts a cubic phase with an *Fm* $\bar{3}$ *m* space group.^[^ ^26^ ^]^ The final product predominantly consists of nanoparticle cubes ≈500 nm in size. In this work, a series of mixed electrolytes was prepared by adding 1 m AlCl_3_ salt to LaCl_3_ solutions of varying concentrations (0, 5, 10, 20, and 50 mm), which were labeled as 0, 5, 10, 20, and 50 L‐A, respectively. Raman spectroscopy and Fourier‐transform infrared spectroscopy (FT‐IR) were used to analyze electrolytes with varying concentrations of LaCl_3_ additives, aiming to investigate their effect on the solvation structure of Al^3+^. The O‐H stretching vibrational modes of water (3000–3700 cm^−1^) can be classified into three types of hydrogen bonding networks: network water (NW) with strong hydrogen bonding at 3230.2 cm^−1^, intermediate water (IW) with moderately strong hydrogen bonding at 3417.9 cm^−1^ and multimeric water (MW) with weak hydrogen bonding at 3552.7 cm^−1^.^[^ ^27^ ^]^ In general, inorganic additives do not significantly affect the solvated structure of metal cations^[^ ^28^ ^]^; however, some studies have shown that halogen anions partially modulate the solvated structure of metal cations.^[^ ^29^, ^30^ ^]^ In the Raman spectroscopy analysis, the bands ≈350 and 525 cm^−1^ are correspond to the symmetric stretching vibrations of Al‐Cl and Al‐O, respectively.^[^ ^31^, ^32^ ^]^ With the increase in additive content, the Al‐Cl peak exhibits a noticeable red‐shift, indicating that the Cl^−^ in the additive influences the solvation structure of Al^3+^ and leads to a change in the vibration frequency (**Figure** **2**a). The results of the FT‐IR tests showed a slight redshift in the spectra as the LaCl_3_ content increased (Figure 2b). Subsequently, the peak area ratios of NW, IW, and MW in different electrolytes were fitted using Gaussian functions (Figure 2c,d; Figure S3, Supporting Information). With the increase in LaCl_3_ content, the proportion of NW slightly decreases, while that of IW increases. Combined with Raman spectroscopy results, these findings indicate that the introduction of LaCl_3_ reconstructs the hydrogen‐bond network in the electrolyte, which in turn affects the migration of metal cations within the electrolyte. To confirm the contribution of LaCl_3_ additives to the solvated structure of Al^3+^, the evolution of the Al^3+^ solvation structure in different electrolyte environments was further analyzed through molecular dynamics (MD) simulations (Figure 2e). The results of the molecular dynamics simulations were analyzed to obtain the radial distribution function (RDF) and coordination numbers (CN) for Al^3+^‐H_2_O and Al^3+^‐Cl^−^ interactions, respectively. Theoretically, in aqueous solution, one Al^3+^ tends to solvate with six H_2_O molecules. In Figure 2f, the RDF peak at ≈1.9 Å represents the first solvation shell of Al^3+^,^[^ ^33^ ^]^ at which point the coordination number of Al^3+^‐H_2_O is 5.7. Meanwhile, the RDF analysis of Al^3+^‐Cl^−^ shows that the coordination number of Al^3+^‐Cl^−^ is 0.3 (Figure 2g). Therefore, in the initial aqueous solution of AlCl_3_, the solvation structure of Al^3+^ in the first shell can be expressed as [Al(H_2_O_5.7_Cl_0.3_)]^2.7+^. With the introduction and gradual increase in LaCl_3_ content, the RDF peak intensity of Al^3+^‐H_2_O at ≈1.9 Å in the 10 L‐A electrolyte undergoes a noticeable decrease. The coordination number of Al^3+^‐H_2_O decreases from 5.7 to 4.9, while the corresponding coordination number of Al^3+^‐Cl^−^ increases from 0.3 to 1.1. At this point, the solvation structure of the Al^3+^ can be expressed as [Al(H_2_O_4.9_Cl_1.1_)]^1.9+^. The effect of the additional Cl^−^ introduced into the solution system on the solvation structure between Al^3+^ and H_2_O became more pronounced as the additive content further increased (Figures S4 and S5, Supporting Information). Figure 2h presents the mean square displacement (MSD) curves of Al^3^⁺ in different electrolyte systems, where the slope of the MSD curves is positively correlated with the diffusion coefficient. Apparently, the diffusion kinetics of Al^3+^ were improved after the introduction of LaCl_3_ in the electrolyte, which will contribute to the electrochemical performance of the battery. This suggests that the halide component in the LaCl_3_ additive plays a crucial role in reconstructing the hydrogen‐bond network in the electrolyte, altering the solvation structure of Al^3^⁺, and enhancing its diffusion kinetics.

### 2.2 Electrochemical Performance

**Figure 3:**
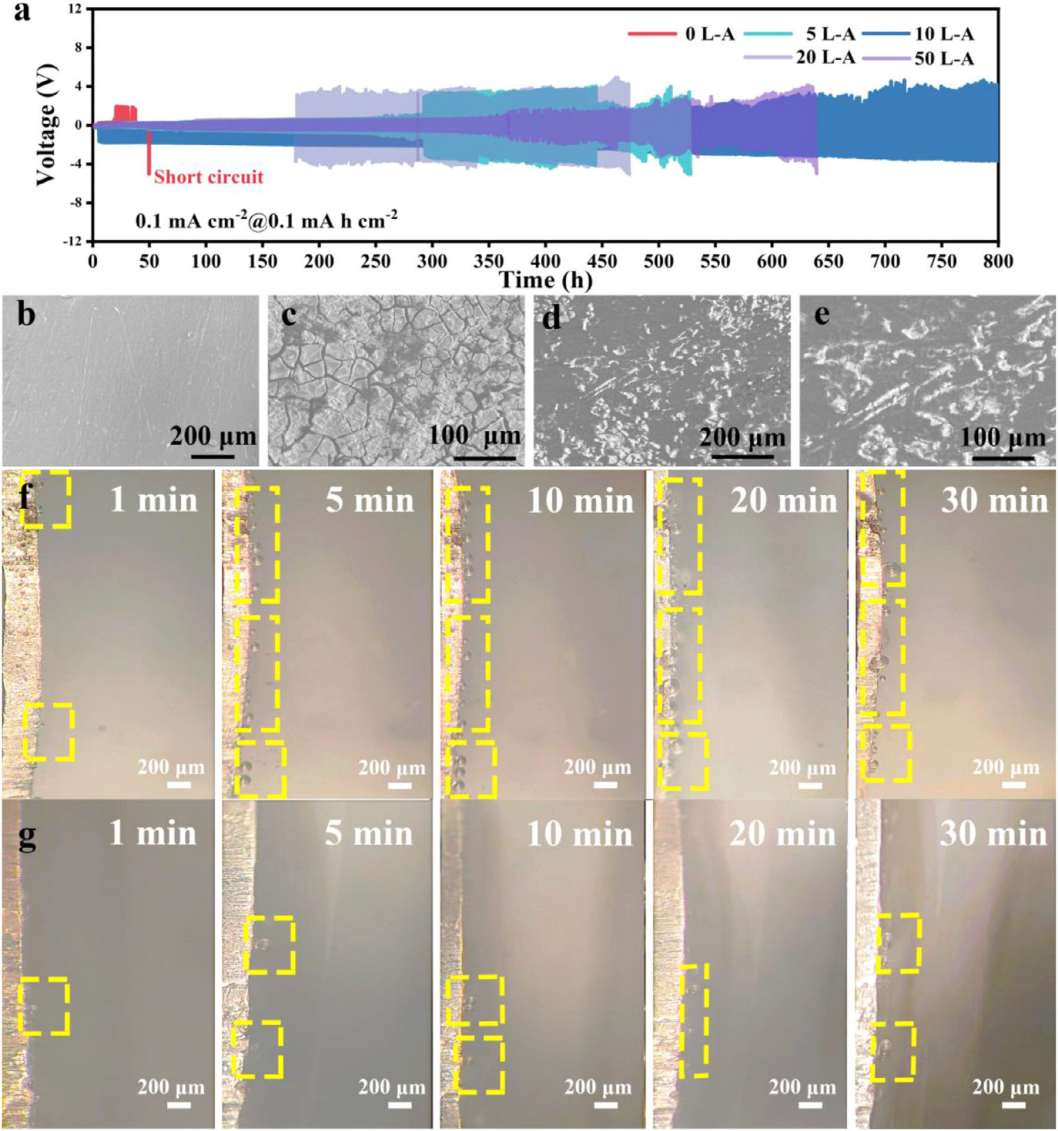
Electrochemical testing of Al//Al symmetric batteries and interfacial behavior of aluminum metal anode under different electrolytes. a) Cycling performance of Al//Al symmetrical batteries in different electrolytes at 0.1 mA cm^−2^ and 0.1 mAh cm^−2^. SEM images of the Al anode in symmetric batteries: b) Initial surface of the Al anode. c) Surface of the Al anode in 0 L‐A electrolyte after 40 h of cycling at 0.1 mA cm^−2^ and 0.1 mAh cm^−2^. d) and e) Surfaces of the Al anode in 10 L‐A electrolyte after 400 h of cycling at 0.1 mA cm^−2^ and 0.1 mAh cm^−2^. In situ optical microscopy images of the behavior of the aluminum‐metal interface after 30 min of symmetric batteries at 0.1 mA cm^−2^ for f) 0 L‐A and g) 10 L‐A electrolytes.

For aluminum‐metal batteries, the stability of the electrolyte is a crucial factor in achieving stable long‐term cycling. Therefore, an Al//Al symmetric batteries were assembled to evaluate the stability of the electrolyte environment during the reaction process. As shown in **Figure** **3**a, the symmetric battery of 0 L‐A at a current density of 0.1 mA cm^−2^ and a capacity of 0.1 mAh cm^−2^ fails after 50 h due to a complete short circuit. Short circuits in Al//Al symmetric batteries are often caused by the aggressive corrosion reaction of the acidic electrolyte on the aluminum metal anode, which leads to gas evolution and triggers the deformation of the batteries. In contrast, a 10 L‐A Al//Al symmetric battery can operate continuously for over 600 h. Therefore, subsequent tests and analyses were carried out with 10 L‐A as the study object. Furthermore, the surface morphologies of the aluminum metal electrodes in Al//Al symmetric batteries tested under different electrolyte conditions were characterized by scanning electron microscopy (SEM). Figure 3b shows the initially smooth aluminum metal electrode. After more than 40 h of cycling, the aluminum surface in the 0 L‐A electrolyte exhibits uneven cracking, indicating obvious corrosion reactions (Figure 3c), followed by a short circuit of the symmetric battery. Figure 3d,e depict the Al electrode surfaces after 400 h of repeated stripping/plating in the 10 L‐A electrolyte at different magnifications. Although corrosion traces are also observed on the aluminum metal surface, it is much denser and flatter compared to the aluminum metal electrode in the 0 L‐A electrolyte. To further assess the stability of the electrolyte in the Al//Al symmetric batteries during the reaction, the corrosion reaction at the aluminum metal interface was monitored In situ using an optical microscope (Figure 3f,g). In situ inspection revealed that bubbles due to the corrosion reaction continuously accumulated on the surface of the aluminum metal anode in the 0 L‐A electrolyte during Al//Al symmetric batteries operation, while only a few bubbles were generated in the 10 L‐A electrolyte. These results indicate that the LaCl_3_ additive effectively mitigates the corrosion reaction and improves the internal environment of the batteries.

**Figure 4:**
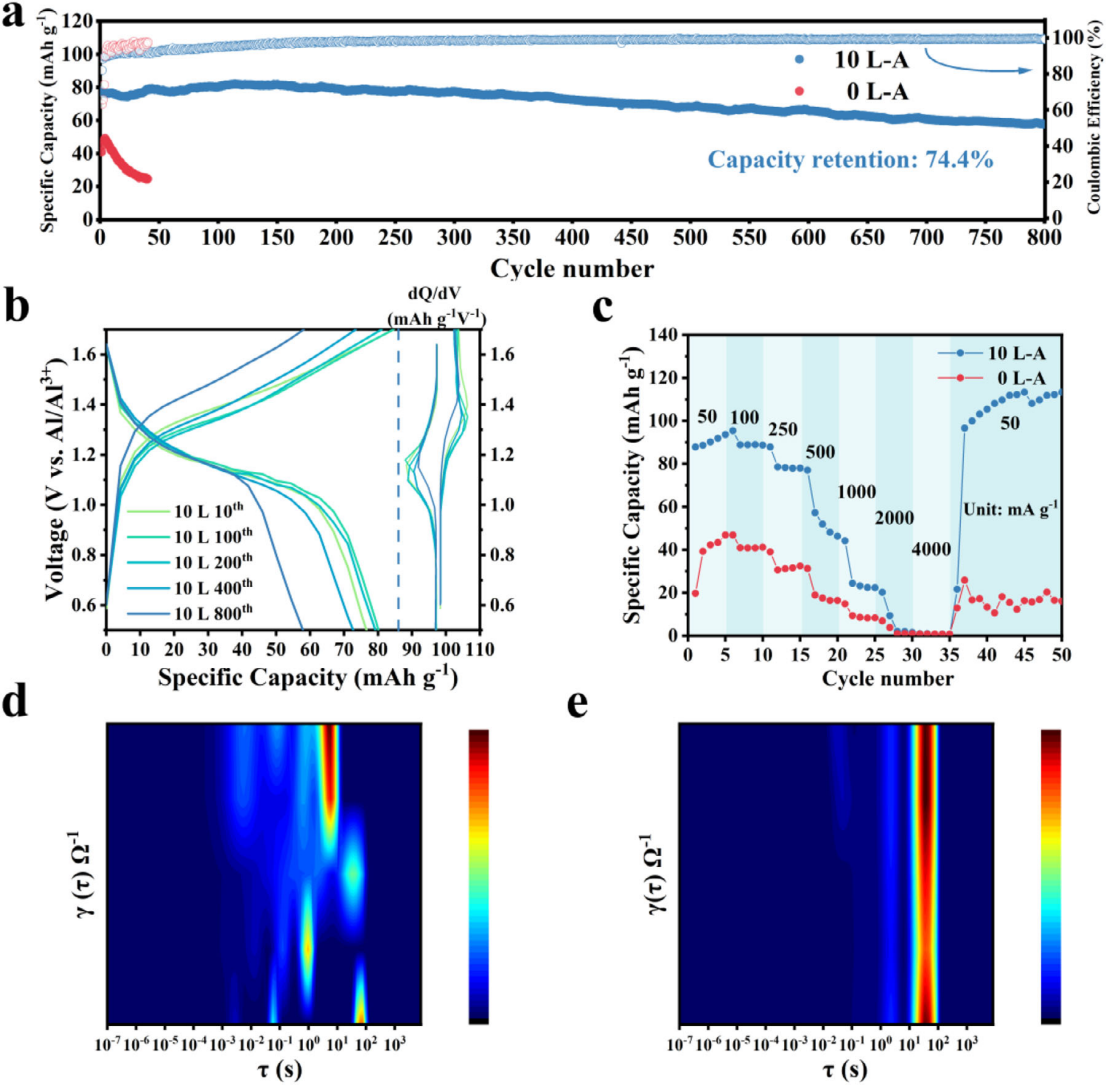
Electrochemical performance of aluminum‐metal full batteries. a) Cycling performance of the full‐batteries with 0 and 10 L‐A full batteries at 250 mA g^−1^. b) Galvanostatic charge/discharge (GCD) curves and corresponding differential capacity vs voltage (dQ/dV) curves for 10 L‐A full battery at a current density of 250 mA g^−1^. c) Rate performance. Evolution of DRT in different electrolyte environments: d) 0 L‐A and e) 10 L‐A full batteries.

To further explore the practical application potential of LaCl_3_ additive in aluminum metal‐based full battery systems, a CoHCF//X L‐A//Al aluminum‐metal batteries were assembled and tested electrochemically (X: 0, 5, 10, 20, and 50). The 10 L‐A full battery in **Figure** **4**a and Figure S6 (Supporting Information) exhibited exceptionally stable cycling performance in constant‐current cycling tests. The initial discharge capacity was 77.7 mAh g^−1^ over the voltage range of 0.5 to 1.7 V at a current density of 250 mA g^−1^. Capacity retention was 87.9% after the first 500 cycles and 74.4% at the 800th cycle, with an average Coulombic efficiency of over 97%. In contrast, the aluminum metal full batteries with 0 L‐A electrolyte exhibited rapid capacity degradation during the cycling test as a result of intense corrosion reactions. The 10 L‐A full battery exhibited excellent cycling stability in constant‐current cycling tests (Figure S7 and Table S1, Supporting Information). Figure 4b and Figure S8 (Supporting Information) show the Galvanostatic charge/discharge (GCD) curves, differential capacity vs voltage (dQ/dV) curves, and cyclic voltammetry (CV) curves of 10 L‐A and 0 L‐A full batteries at different cycling numbers. The test results indicate that the 10 L‐A electrolyte provides a stable reaction environment for the reversible intercalation of Al^3+^. Figure 4c shows rate performance tests for current densities from 50 to 4000 mA g^−1^. The discharge capacities of 10 L‐A full battery at current densities of 50, 100, 250, 500, and 1000 mA g^−1^ were 93.6, 88.7, 46.3, and 22.3 mAh g^−1^, respectively (values taken are the final discharge capacity values for each current density tested). The discharge capacity of all batteries fell below 1 mAh g^−1^ when the current density was increased to 2000 and 4000 mA g^−1^. The discharge capacity of 10 L‐A full battery recovered to 113.3 mAh g^−1^ when the test current density was brought back to 50 mA g^−1^. The results of both cycling and rate tests confirmed that the electrochemical performance of the aluminum‐metal full batteries was visibly improved by the addition of LaCl_3_ to the AlCl_3_ electrolyte.

To further investigate the enhanced cycling stability with LaCl_3_ addition, electrochemical impedance spectroscopy (EIS) and distribution of relaxation time (DRT) analysis were conducted for 0 L‐A and 10 L‐A full batteries during the first 100 cycles. As shown in Figure S9 (Supporting Information), during the preparation stage of the battery (0th cycle) and the initial cycling stage (10th cycle), the impedance spectrum of a 0 L‐A full battery exhibits a noticeable inductive arc in the fourth quadrant of the low‐frequency region. As the cycling progresses, the inductive arc is gradually replaced by a second capacitive arc. However, no similar phenomenon was observed in the impedance spectrum of 10 L‐A full battery (Figure S10, Supporting Information). Furthermore, the DRT analysis (Figure 4d,e) reveals that the introduction of LaCl_3_ into the electrolyte improves the interfacial reaction stability and reversibility. The EIS spectrum of 0 L‐A full battery typically reflects two stages of the interfacial reactions of aluminum metal in the chloride‐containing acidic system: the initiation stage of pitting corrosion (0th to 10th cycle) and the development stage (10th to 100th cycle).^[^ ^34^, ^35^ ^]^ In the early cycling stage of 0 L‐A (0th to 10th cycles), Cl^−^ adsorption occurs on the aluminum metal surface, and the impedance spectrum of 0 L‐A actually consists of two regions, the non‐Cl^−^ adsorption region and the Cl^−^ adsorption region. Therefore, the impedance spectrum of 0 L‐A takes the form of a composite of capacitive and inductive arcs. As the electrochemical cycle progresses, the susceptibility arc gradually decreases. After the 50th cycle, Cl^−^ completes the vertical dissolution penetration at the aluminum‐metal interface in its adsorption area, forming a corrosion pit. As a result, the impedance spectrum changes to a double capacitive arc pattern consisting of Faraday impedance in the passivation film region and Faraday impedance in the corrosion pit region. Although Cl^−^ from LaCl_3_ in the 10 L‐A hybrid electrolyte partially acts as an additional halogen anion that modulates the solvated structure of Al^3+^, it remains the culprit for interfacial disruption and corrosion of the aluminum metal anode in the electrolyte.

### 2.3 Aluminum Metal Interface Characterization and Mechanism Analysis

**Figure 5:**
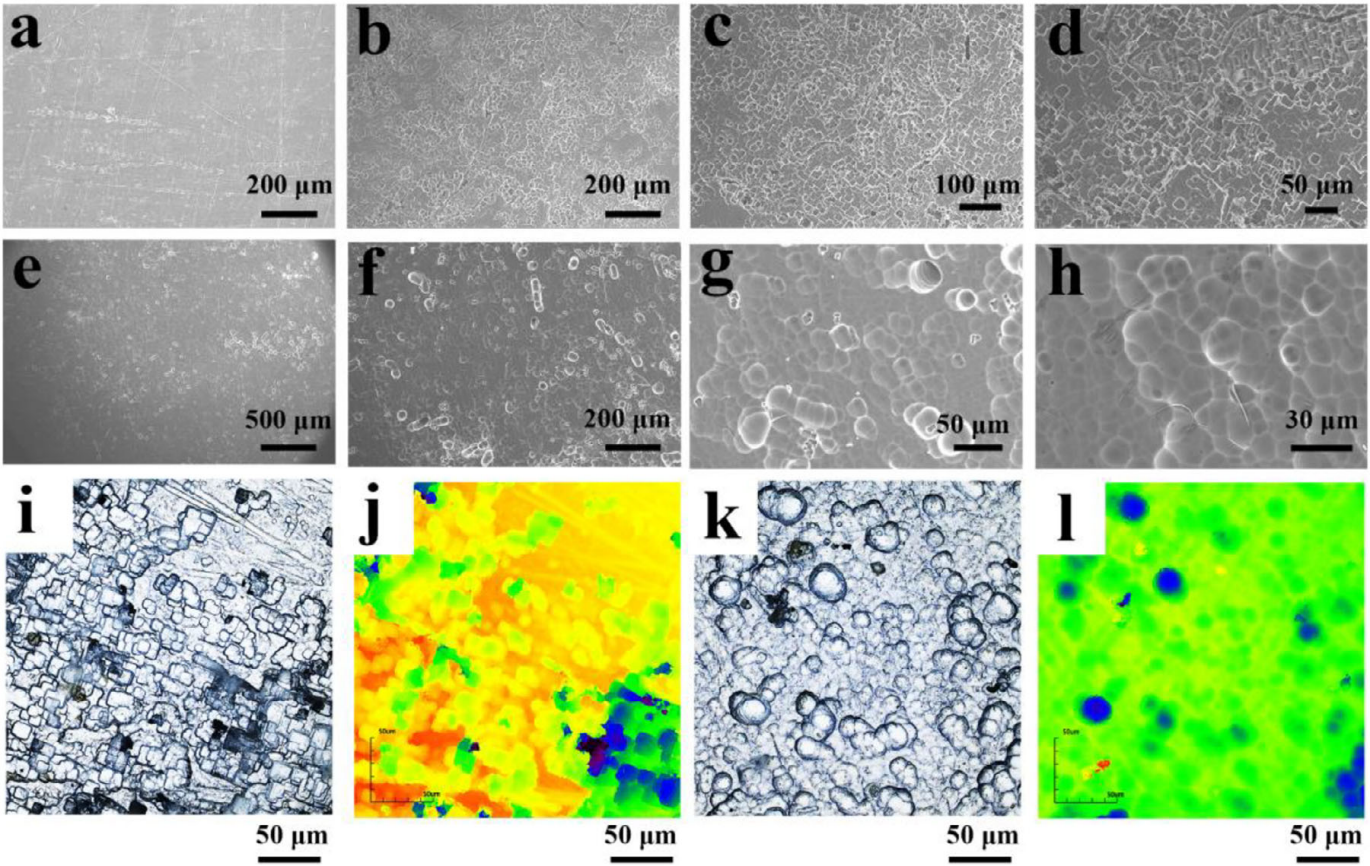
Characterization of the aluminum‐metal anode interface. a) Original aluminum metal interface. b–d): SEM images of aluminum metal anode electrodes after 800th cycling in 0 L‐A electrolyte at different scales. e–h): SEM images of aluminum metal anode electrodes after 800th cycling in 10 L‐A electrolyte at different scales. i–l) 2D CLSM images of aluminum metal anode electrodes with different electrolytes after 800 cycles: i,j): 0 L‐A; k) and l): 10 L‐A.

To further explore the working mechanism of the battery, SEM and confocal laser scanning microscopy (CLSM) were employed to characterize the interfaces of aluminum metal anodes after cycling in different electrolyte environments and to analyze the correlation between aluminum metal interfaces and cycling stability. **Figure** **5**a shows the initial aluminum metal anode interface. Figure 5b–d displays the interfacial morphology of the aluminum metal at different scales after 800 cycles in the 0 L‐A electrolyte. It can be observed that the aluminum metal surface is densely covered with pitting corrosion, and there is also evidence of surface cracking on the aluminum metal. Figure 5e–h shows the interfacial morphology of aluminum metal after 800 cycles in 10 L‐A electrolyte, and the integrity of the aluminum metal surface sharply contrasts with that observed in 0 L‐A electrolyte (Figure 5e). Continuous magnification and observation of the aluminum metal surface in 10 L‐A electrolyte revealed that the interfacial morphology was predominantly characterized by a combination of rounded and smooth continuous corrosion forms (Figure 5f–h; Figure S11, Supporting Information). At the same time, a small number of pores were also observed on the dense surface (Figure S12, Supporting Information). The morphology and roughness of the aluminum metal surface in different electrolyte environments were next further evaluated and quantified by CLSM. The 2D and 3D CLSM images for 0 L‐A and 10 L‐A are shown in Figure 5i–l and Figure S13a,b (Supporting Information), respectively. After the introduction of LaCl_3_ into the electrolyte, the aluminum metal surface after long cycling does not show a noticeable interfacial height difference. The roughness profile indicates a marked reduction in the aluminum‐metal interface roughness for 10 L‐A (Figure S14b, Supporting Information, R_z_ = 0.358 µm) compared to the 0 L‐A aluminum‐metal anode (Figure S14a, Supporting Information, R_z_ = 2.591 µm). Through a series of characterizations of the aluminum metal anode surface, it is observed that the interface of the aluminum metal anode, after the introduction of La^3+^ into the AlCl_3_ electrolyte, undergoes a transition from a sharp, rough localized corrosion morphology to a more uniform corrosion morphology with a smoother interface.

**Figure 6:**
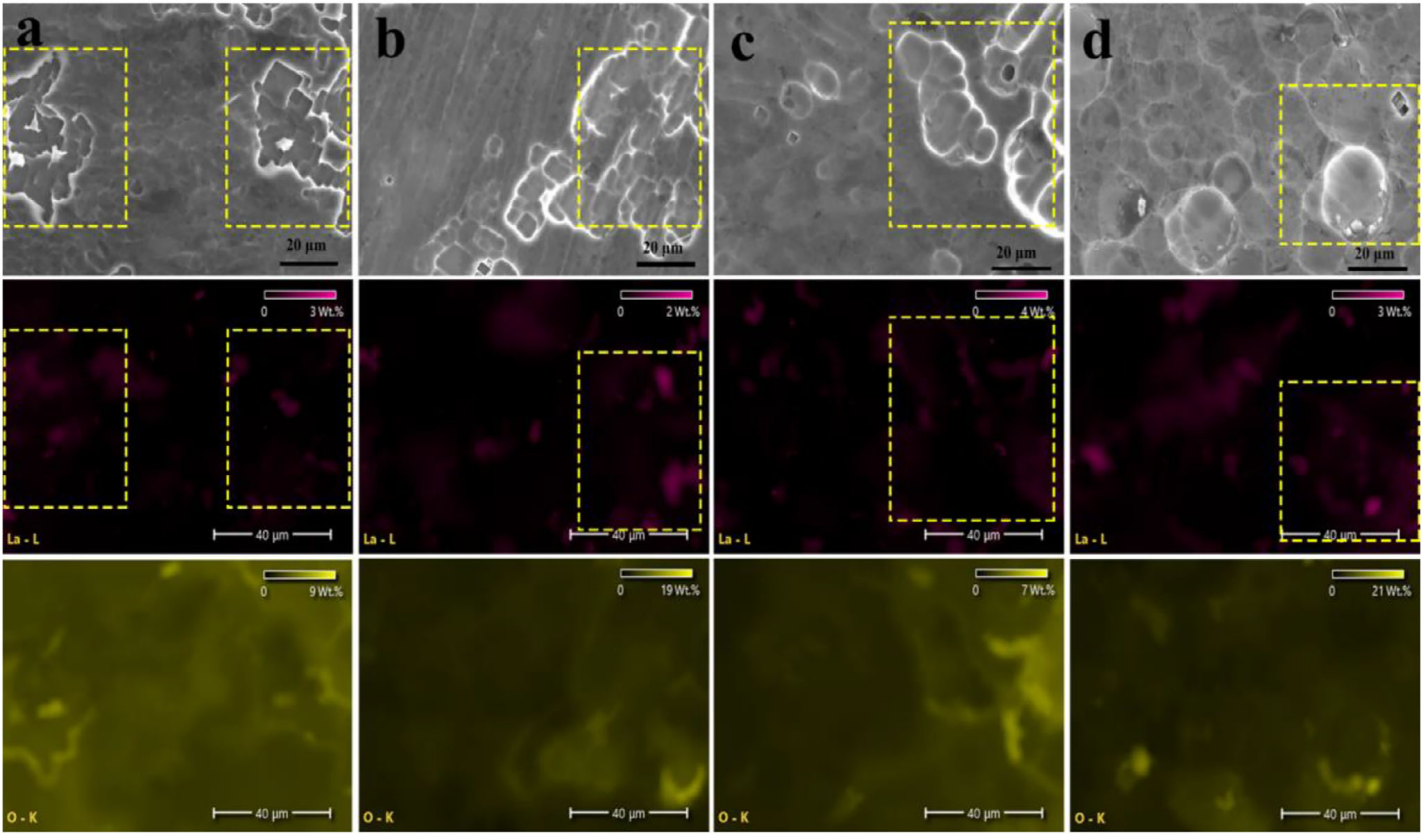
EDS spectra of 10 L‐A aluminum metal anode with different numbers of cycle turns. a) 100th. b) 200th. c) 500th and d) 800th.

**Figure 7:**
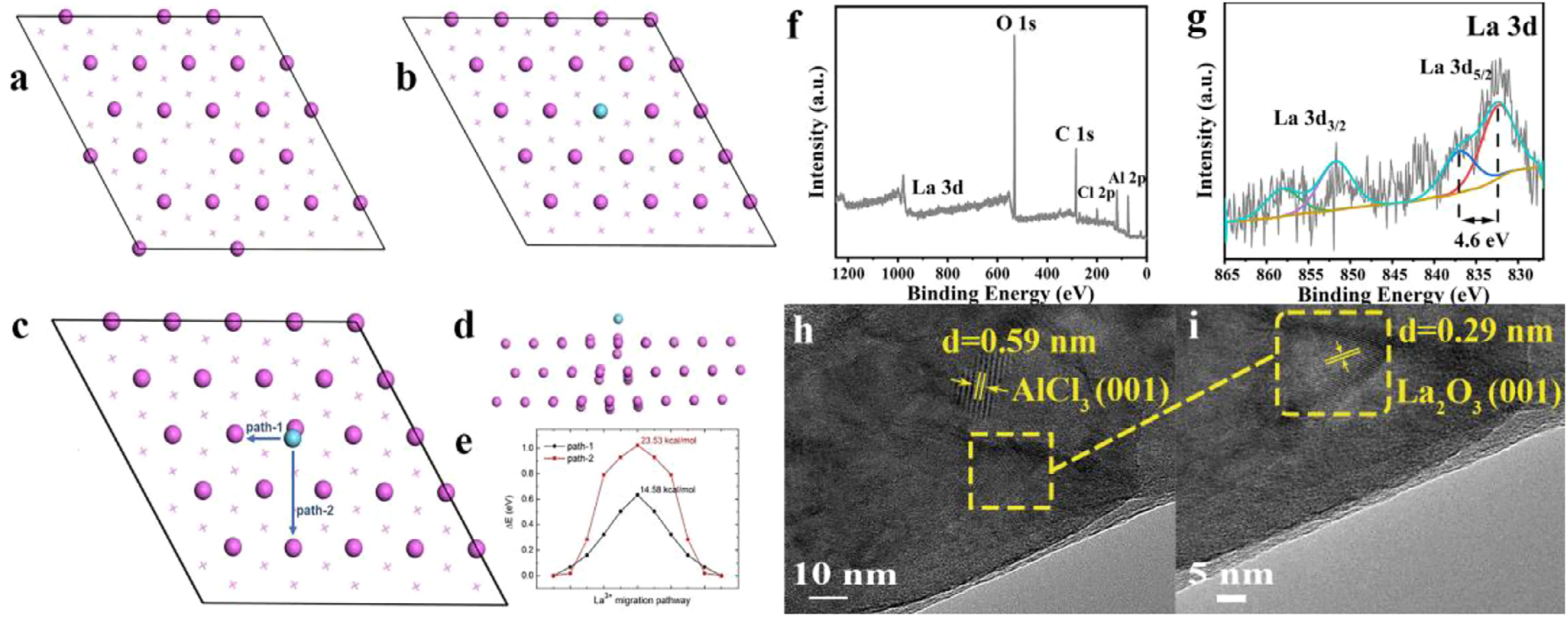
DFT calculations and material characterization of 10 L‐A aluminum metal anode after 800 cycles. a) Models of Al (111) crystal surface with aluminum vacancies. b) Models of La^3+^ adsorption on Al (111) crystal surface with aluminum vacancies. c) Migration paths of La^3+^ on a perfect Al (111) crystal surface. d) Surface remodeling induced by La^3+^ on a perfect Al (111) crystal surface. e) migration energy barriers for different migration paths. f) XPS full scan spectrum and g) La 3d deconvolution. HR‐TEM micrographs of a 10 L‐A aluminum metal anode after 800 cycles sampled by FIB. h) Low and i) high magnification. The lattice spacing indicated by the yellow arrows in (i) is 0.29 nm.

To analyze the function of La^3+^ during electrochemical cycling, Energy Dispersive X‐ray Spectroscopy (EDS) tests were performed on the surface of the aluminum metal anode in the 10 L‐A electrolyte after different numbers of cycles (**Figure** **6**a–d). Interestingly, the EDS results revealed that lanthanum elements are predominantly concentrated and clustered around the areas where corrosion traces are observed on the aluminum metal anode surface (highlighted by the yellow dashed rectangle in the figures). In contrast, lanthanum elements are noticeably more sparsely distributed in areas without visible corrosion. To further corroborate this phenomenon, EDS characterization was subsequently performed on the aluminum electrode surface in Al//Al symmetric batteries containing 10 L‐A and higher additive concentrations. Compared to full battery tests, the symmetric battery's reaction environment, combined with higher additive concentration and repeated Al surface plating/stripping, better facilitates the characterization of La enrichment tendency. Figure S15a–c (Supporting Information) presents EDS results of Al electrodes in cycled Al//Al symmetric batteries with different additive‐containing electrolytes. The results indicate that La preferentially accumulates at corroded sites on the Al electrode surface, consistent with its aggregation tendency in full batteries. In order to further analyze the lanthanide enrichment phenomenon in the EDS experiment, the adsorption behavior of La^3+^ on the aluminum metal surface was evaluated by Density Functional Theory (DFT) calculations. Therefore, Al (111) crystal surface with Al vacancies and a perfect Al (111) crystal surface were constructed (**Figure** **7**a). As shown in Figure 7b, the adsorption of La^3+^ on the defective Al (111) surface does not cause significant structural distortion, whereas its adsorption on the perfect Al (111) surface leads to a more noticeable local reconstruction (Figure 7d). According to the calculation based on Equation (7), the difference in adsorption energies between the two types of Al (111) surfaces is ≈−0.777 eV, which indicates that La^3+^ prefers to adsorb on defective aluminum metal surfaces. In addition, DFT calculations were carried out to further evaluate the migration energy barrier of La^3+^ on the perfect Al (111) surface. Two migration pathways were considered (Figure 7c), and the corresponding energy barrier was calculated to be ≈0.634 eV (14.582 kcal mol^−1^, Figure 7e), suggesting that La^3+^ is capable of migrating across the Al surface and reaching defect sites, which are generally more susceptible to corrosion. The elemental composition and chemical valence states of the cycled aluminum metal surface were further analyzed using X‐ray Photoelectron Spectroscopy (XPS, Figure 7f). Figure 7g shows the XPS spectra of La 3d, exhibiting two components at ≈835 and 853 eV for La 3d_5/2_ and La 3d_3/2_, respectively. The key feature is the multiple splitting of each component, the magnitude of La 3d_5/2_ multiple splitting is ≈4.6 eV, which suggests the formation of La_2_O_3_ on the aluminum metal anode surface after electrochemical cycling.^[^ ^36^ ^]^ Figure S16a (Supporting Information) also shows the Al 2p XPS spectrum, which can be straightforwardly deconvoluted into AlCl_3_/Al_2_O_3_ and Al° components, with binding energies around of 75 and 72.3 eV, respectively.^[^ ^37^, ^38^ ^]^ The XPS spectrum of Cl 2p (Figure S16b, Supporting Information) shows two distinct peaks near 199.2 and 201 eV, corresponding to the Cl 2p_3/2_ and Cl 2p_1/2_ signals of Cl^−^.^[^ ^39^ ^]^ Comprehensive analyses by EDS, DFT, and XPS indicated that during the reaction process of the aluminum metal anode, La^3+^ in the electrolyte would be enriched in the corrosion‐prone defects on the surface of the aluminum metal and eventually distributed and aggregated as lanthanum oxides. In fact, an amorphous passivation film inevitably forms on the surface of the aluminum anode during the entire electrochemical process. To further investigate the mechanism of La_2_O_3_ function, the surface of the 10 L‐A aluminum metal anode after cycling was cut and sampled using a Focused Ion Beam (FIB) and observed by High‐Resolution Transmission Electron Microscopy (HR‐TEM). The HR‐TEM results of the localized magnification test of Figure 7h are shown in Figure 7i. The collected samples exhibit two distinct lattice diffraction patterns with different widths on their surfaces. The measured lattice spacings are 0.59 and 0.29 nm, respectively. The 0.59 nm lattice spacing corresponds to the (001) crystal plane of AlCl_3_ (JCPDS 22‐0010). The AlCl_3_ on the aluminum metal anode surface may result from the gradual decomposition of the aqueous electrolyte solvent and the subsequent precipitation of the solute during long‐cycle processes. The 0.29 nm lattice spacing corresponds to the (101) crystal plane of La_2_O_3_.^[^ ^40^ ^]^

**Figure 8:**
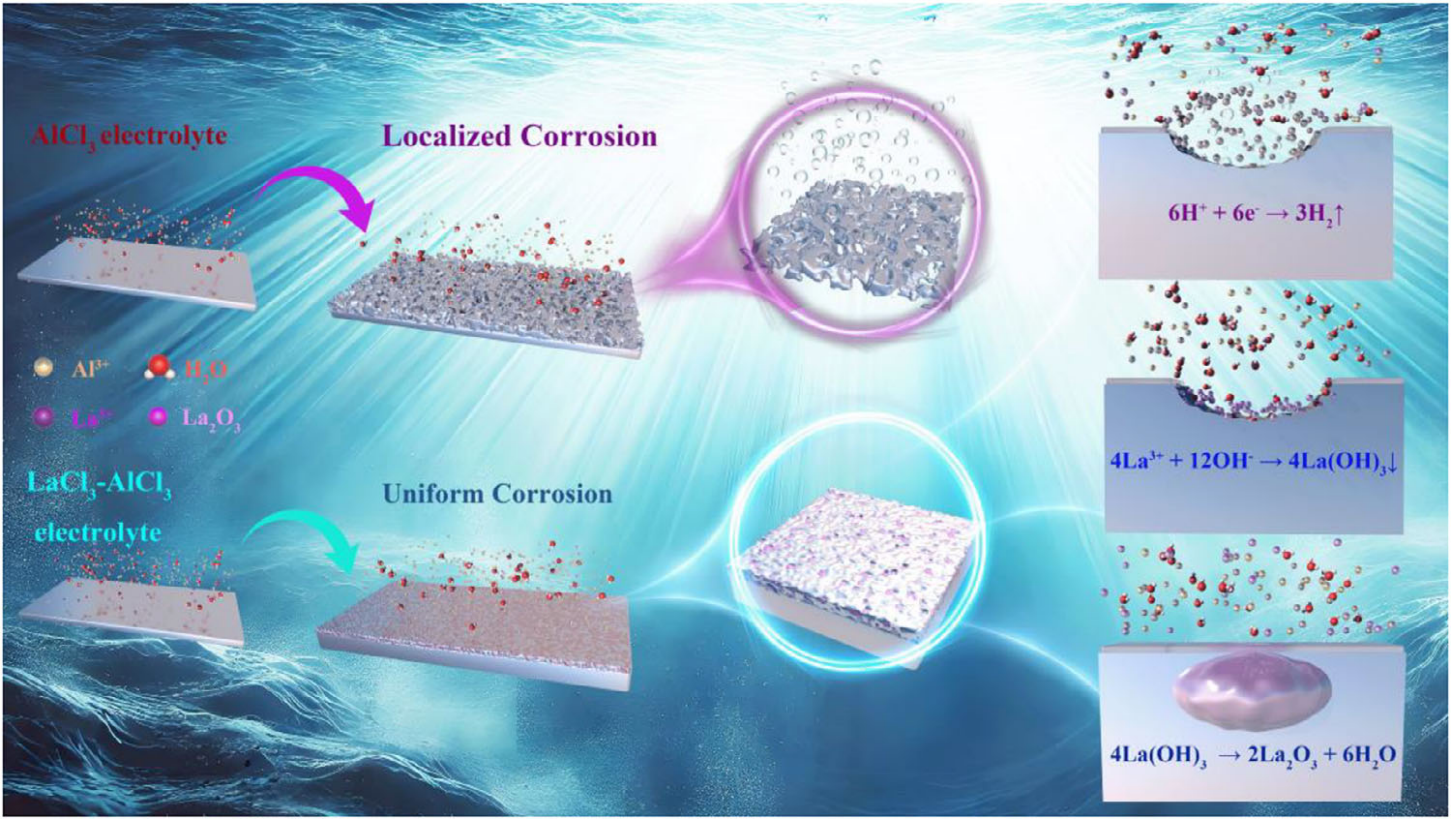
Schematic diagram of rare earth additives mitigating interfacial reactions at the aluminum‐metal anode.

Based on the surface analysis of the aluminum metal anode, the reaction mechanism is proposed as illustrated in **Figure** **8**. Due to the inherent inhomogeneity at the interface between aluminum metal and the oxide film, the local corrosion rate of Cl^−^ varies across the interface.^[^ ^41^ ^]^ Cl^−^ preferentially attacks defective regions of the oxide film, initiating localized corrosion that progresses vertically, penetrating and expanding over time. As the oxide film deteriorates, the exposed aluminum metal comes into direct contact with the corrosive electrolyte, leading to rapid cation release accompanied by gas evolution. The following reactions mainly occur during this period^[^ ^4^, ^42^ ^]^:

(1) $$2Al\;\to \;2Al^{3+}+6e^{-}$$

(2) $$6H^{+}+6e^{-}\;\to \;3H_{2}\;↑$$

The intense corrosion reaction leads to the rapid depletion of a large amount of electrolyte. Meanwhile, the continuous gas production exerts a severe negative impact on the available volume within the battery. The ongoing accumulation of these detrimental factors results in the progressive deterioration of the battery's cycling performance, continuing from the onset of the test until the electrolyte is completely exhausted. Upon the introduction of La^3+^ ions into the electrolyte, Cl^−^ ions still preferentially attack the defect sites at the interface of the passivation film on the aluminum surface during the initial stage of corrosion. As the passivation layer is gradually damaged, more bare aluminum is exposed to the electrolyte. However, La^3+^ ions in the electrolyte tend to adsorb and accumulate at the defect‐rich regions on the aluminum surface that are susceptible to corrosion, as supported by DFT calculations. Once corrosion initiates at the Al‐electrolyte interface, localized pH changes occur. The electrochemically inert La^3+^ ions enriched near these corrosion sites react with OH^−^ to form La(OH)_3_, which subsequently transforms into La_2_O_3_. The possible reaction equations are as follows^[^ ^43^, ^44^, ^45^ ^]^:

(3) $$2Al\;\to \;2Al^{3+}+6e^{-}$$

(4) $$O_{2}+6H_{2}O+12e^{-}\;\to 12OH^{-}$$

(5) $$4La^{3+}+12OH^{-}\;\to \;4La{\left(OH\right)}_{3}\;↓$$

Eventually transforms into lanthanum oxide:

(6) $$4La{\left(OH\right)}_{3}\;\to \;2La_{2}O_{3}+6H_{2}O$$

The precipitates formed by rare earth ions preferentially deposit on corrosion‐prone sites of the Al surface, as confirmed by EDS mapping. This deposition reduces the exposure of highly active aluminum areas to the corrosive environment, thereby shifting the corrosion behavior from aggressive, localized corrosion to a slower and more uniform mode. Consequently, the surface morphology of the Al anode evolves from inhomogeneous pitting to a smooth, continuous, and uniform corrosion pattern. Guided by La^3^⁺ ions, parasitic reactions at the aluminum anode are effectively suppressed, contributing to the stabilization of the battery system and significantly enhancing its cycling stability.

**Figure 9:**
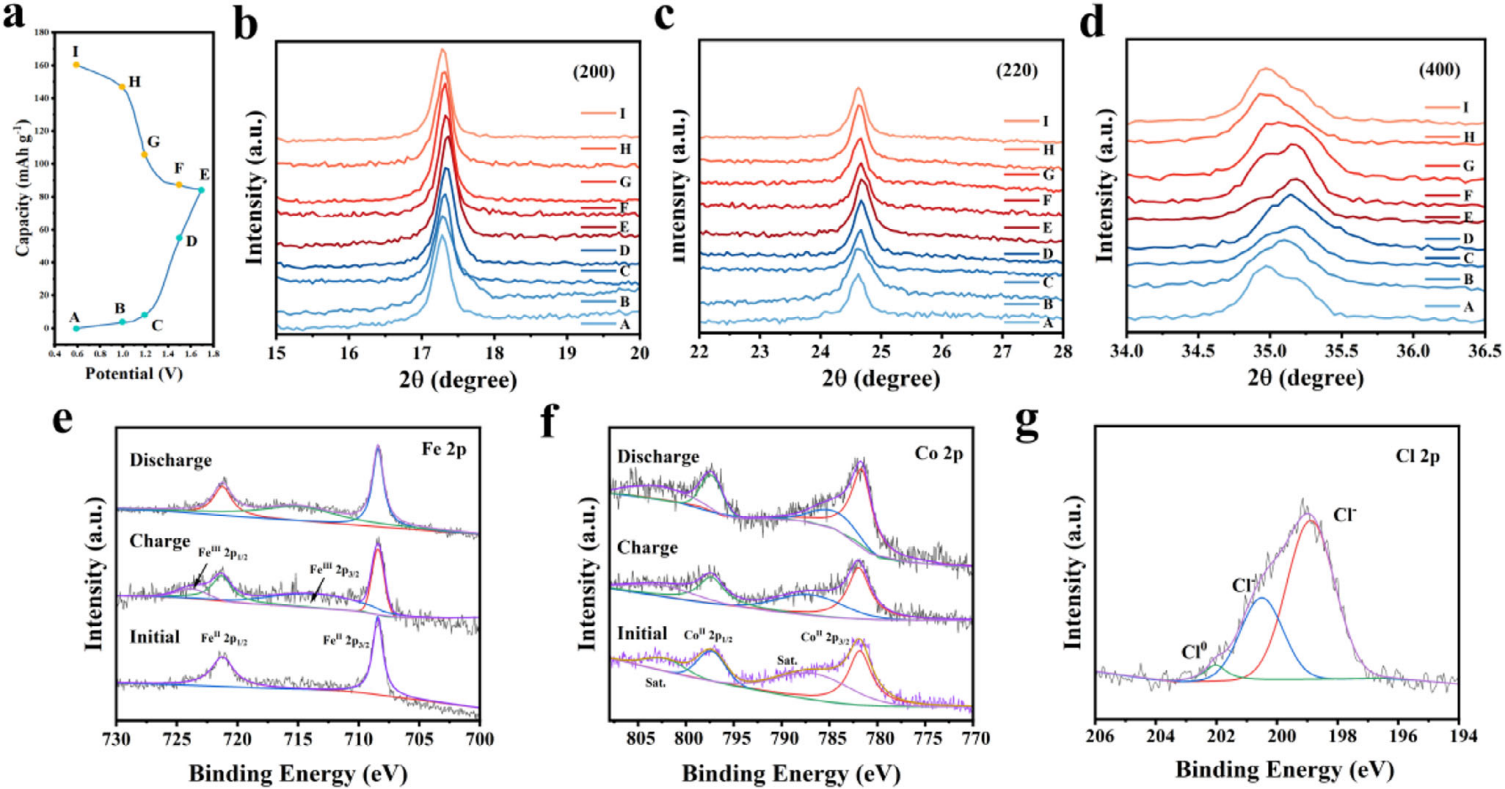
Study on the energy storage mechanism of CoHCF//Al full batteries. a) Charge–discharge curve of CoHCF cathode at a current density of 250 mA g^−1^. b–d) Ex situ XRD patterns corresponding to the characteristic crystal planes of CoHCF under different charge‐discharge states in Figure 9a. Ex situ XPS analysis of Fe 2p e), Co 2p f) and g) Cl 2p Ex situ XPS spectra of the CoHCF cathode charged to the high potential (1.7 V).

Subsequently, the energy storage mechanism of the CoHCF//Al full batteries was investigated using Ex situ XRD and Ex situ XPS. The Ex situ XRD (**Figure** **9**a–d) results demonstrate that during the charging process, the (200), (220), and (400) diffraction peaks of the CoHCF crystals distinctly shift toward higher angles, while the peak positions gradually revert to their initial values during the discharging process. This reflects the reversible expansion and contraction of the crystal structure of the cathode material during the Al^3+^ intercalation and deintercalation reactions, which is conducive to the cyclic stability of the battery. The Ex situ XPS results show no valence state change of Al^3+^ during charge–discharge, confirming the intercalation/deintercalation mechanism between Al^3+^ and CoHCF (Figure S17, Supporting Information). Furthermore, Fe^2+^ in CoHCF is oxidized during the charging process, and the Fe^3+^ peak gradually disappears upon discharging (Figure 9e); however, the valence state of Co does not exhibit noticeable change throughout the charge–discharge cycle (Figure 9f). Notably, Ex situ XPS of Cl in the 1.7 V charged cathode material shows that besides Cl^−^, a Cl─Cl bond‐related peak appears at 201.6–202 eV (Figure 9g). This suggests that in addition to the Fe^2^⁺/Fe^3^⁺ redox couple, the Cl^−^/Cl^0^ redox couple exists, particularly at high potentials.^[^ ^26^, ^46^ ^]^

## 3 Conclusion

In conclusion, we introduced a trace amount of LaCl_3_ into the inexpensive yet highly corrosive aqueous AlCl_3_ solution as the electrolyte for aqueous aluminum‐metal batteries. While the introduced halogen anion (Cl^−^) regulates the solvated structure of Al^3+^, the electrochemically inert rare earth ion, La^3+^, plays a more critical role by forming protective precipitates at the corrosion sites during the battery's reaction process. This helps to mitigate the destructive effects of the acidic electrolyte on the aluminum‐metal‐anode interface, stabilizing the internal volume space and reaction environment of the battery system. As a result, the 10 L‐A aluminum‐metal full battery achieved a stable performance at 250 mA g^−1^ for 800 cycles, with a capacity retention of 74.4% and an average Coulombic efficiency exceeding 97%. This study offers a promising reference for the future application of aqueous aluminum‐metal battery systems utilizing inexpensive aqueous AlCl_3_ solution as the electrolyte. It also paves the way for the development of metal anticorrosion science.

## 4 Experimental Section

### Preparation of Electrolytes

The electrolyte was prepared by dissolving 1 m AlCl_3_ and various concentrations of LaCl_3_ (LaCl_3_ 0, 5, 10, 20, and 50 mm) in deionized water. The amount of electrolyte used in both the Al//Al symmetric and aluminum‐metal full battery tests was 80 µL.

### Preparation of CoHCF Prussian Blue Analogs Cathode Materials

CoHCF cathode materials were synthesized by a typical co‐precipitation method.^[^ ^26^ ^]^ First, 0.2 mmol of K_3_[Fe(CN)_6_] and 1.4 g of sodium dodecyl sulfate (SDS) were dissolved in 40 mL of deionized water to obtain solution A. Then, 4 mmol of Co(CH_3_COO)_2_·4H_2_O was dissolved in 40 mL of deionized water to obtain solution B. Then, Solution B was added to Solution A slowly and dropwise to produce a brownish–yellow precipitate, and after the dropwise addition, the mixed solution was aged at room temperature for 24 h, and the precipitate was collected by centrifugal washing. Finally, the resulting precipitate was dried in a vacuum oven at 70 °C overnight.

### Anode Material Pretreatment

Aluminum metal foil (99.9% purity, 0.25 mm thickness), polished with sandpaper and ultrasonically cleaned for half an hour before use, and cut the aluminum metal into round electrode plates with a diameter of 11 mm when used.

### Preparation and Testing of CoHCF Cathode Electrodes and Aluminum‐Metal Full Battery Assembly

Preparation of CoHCF cathode electrodes: CoHCF powder was mixed with carbon black and polyvinylidene fluoride (PVDF, mass ratio: 7:2:1) in *N*‐Methylpyrrolidone (NMP) to obtain a slurry, which was then coated onto the titanium foil. It was dried in a vacuum oven at 100 °C for 12 h and then cut into an electrode of 10 mm diameter and set aside. Full battery assembly and testing: Electrochemical testing using a two‐electrode Swagelok battery. The anode materials were made of pretreated metallic aluminum foil, and a piece of glass microfiber was placed between the cathode and the anode as a diaphragm (Whatman GF/A, 13 mm diameter). Cycle performance and rate tests were performed using the LAND Battery Test System over a voltage range of 0.5–1.7 V (vs Al/Al^3+^). The batteries were subjected to cyclic voltammetry (CV) and electrochemical impedance spectroscopy tests using an EC‐LAB VMP3 electrochemical workstation. The CV tests were performed at a scan rate of 0.2 mV s^−1^. The electrochemical impedance spectroscopy (EIS) tests were performed over a frequency range of 1 MHz to 0.01 Hz. All electrochemical tests were performed at room temperature.

### Assembly and Test of Al//Al Symmetrical Batteries

Aluminum symmetrical batteries were also tested with a two‐electrode Swagelok battery mold with pretreated aluminum metal foil on both sides of the electrodes. Constant charge/discharge cycles were performed at 0.1 mA cm^−2^@0.1 mAh cm^−2^ current density and capacity using the LAND Battery Test System. In situ optical observations were made using a two‐electrode battery mold (Al//Al symmetrical batteries). The symmetric battery electrolyte content used for the observations was 150 µL, and was tested at a current density of 0.1 mA cm^−2^.

### Characterization of Materials

X‐ray diffraction (XRD) patterns of CoHCF were recorded using an Aeris diffractometer (Malvern Panalytical) and Cu Kα radiation. Field emission scanning electron microscopy (SEM, Thermoscientific Apreo 2, 20 kV) and Energy Dispersive Spectroscopy (EDS, Thermo Scientific ChemiSEM) were used to observe the morphology of the samples and the distribution of elements on the surface. The Raman spectra were collected on a Renishaw micro‐Raman spectrometer (laser wavelength: 532 nm). Fourier transform infrared (FT‐IR) spectra were measured with a Nicolet iS5 (Thermo Fisher Scientific). A VM‐2300 metallographic microscope was used for in situ observation of symmetric batteries, and a confocal laser scanning microscopy (CLSM, Olympus OLS 5100) was used to evaluate the 2D and 3D morphology of the surface of the aluminum metal anode material. X‐ray photoelectron spectroscopy (XPS) experiments were carried out under the base pressure of 2 × 10^−10^ mbar with ESCA 200 electron analyzer and a monochromatic Al kα x‐ray source (hv = 1486.6 eV). All the spectra were collected at normal emission and room temperature. The spectrometer was well calibrated by a sputter‐cleaned Au film with the Fermi level at 0 eV and Au 4f_7/2_ peak at 84.0 eV, with its full width at half maximum being 0.65 eV. The powder sample was prepared on a highly conductive tape with very fine particles after blowing out any superfluous powder for the XPS measurements. HR‐TEM experiments were performed in an HR‐TEM JEOL ARM‐200F with an operating voltage of 200 kV, in addition to cutting the aluminum metal anode after electrochemical cycling by using a focused ion beam (FIB) JEOL JIB‐4000.

### DFT Calculations

First‐principles calculations based on density functional theory (DFT) were carried out using the Vienna Ab initio Simulation Package (VASP).^[^ ^47^ ^]^  The projector augmented‐wave (PAW) method was used to describe core–valence interactions,^[^ ^48^ ^]^ and the Perdew–Burke–Ernzerhof (PBE) functional within the generalized gradient approximation (GGA) was employed for the exchange–correlation energy.^[^ ^49^ ^]^ A plane‐wave cutoff energy of 500 eV was applied. For bulk Al, a 25 × 25 × 25 Monkhorst–Pack k‐point mesh was used. The Al (111) crystal surface was modeled using a 5 × 5 × 1 supercell consisting of three atomic layers and a vacuum slab of 20 Å to avoid spurious interactions between periodic images. A 5 × 5 × 1 k‐point mesh was applied for surface calculations. After full geometric optimization, the relaxed lattice constant of Al was 2.788 Å, and the interlayer spacing between adjacent Al layers was 2.308 Å. A single La^3+^ ion was placed on both the perfect Al (111) crystal surface and a defective surface containing a single Al vacancy within the same supercell, in order to investigate its adsorption behavior. The difference in adsorption energy of La^3+^ on different types of crystal surfaces is calculated as follows:

(7) $$\Delta E_{ads}\left(La^{3+}\right)=\left(E_{vac+La}\;-\;E_{vac}\right)\;-\;\left(E_{perf+La}\;-\;E_{perf}\right)$$

E_perf_ in Equation (7) represents the surface energy of the perfect Al (111) crystal surface and E_vac_ represents the indicated energy of the vacant Al (111) crystal surface.

### MD Simulation

All MD simulations were performed using GROMACS 2020.3 to study the solvated structure of the electrolyte.^[^ ^50^, ^51^ ^]^ The Amber99SB force field was used. The electrolyte was placed in a periodic cubic box in which the NPT set (constant number of particles, temperature, and pressure) was employed. The temperature and pressure were coupled through a V‐rescale thermostat (298.15 K) and a Parrinello–Rahman barometric regulator (reference pressure of 1 bar).^[^ ^52^, ^53^ ^]^ The system reached full equilibrium after 2 ns of simulation, followed by an additional 20 ns run. Throughout the simulation, a time step of 2 fs was maintained, and data were collected for statistical analysis during the final 10 ns. All computational structures in the MD simulations were analyzed using VMD, and radial distribution functions were calculated.^[^ ^54^ ^]^

## Conflict of Interest

The authors declare no conflict of interest.

## Supporting information



Supporting Information

## Data Availability

The data that support the findings of this study are available from the corresponding author upon reasonable request.
