# Supplementary material for: Enhancing Cycling Stability of Aqueous Aluminum‐Metal Batteries via LaCl3‐Modulated Interfacial Reactions
Source: Adv Sci (Weinh). 2025 Dec 19;13(11):e14322. doi: 10.1002/advs.202514322 (PMC12931220; doi:10.1002/advs.202514322)
Supplement: Supplementary file 1 — Supporting Information [file ADVS-13-e14322-s001.docx]

Supporting Information

**Enhancing Cycling Stability of Aqueous Aluminum-Metal Batteries via LaCl_3_-Modulated Interfacial Reactions**

*Yanshen Gao,^a^ Karol Załęski,^b^ Emerson Coy,^b^ Błażej Scheibe,^b^ Kacper Szymański,^a,c^ Qingshan Yang,^a^ Ewa Mijowska,^a,c^ Xianjie Liu,^d^* Ran Jia,^e^*** Dariusz Moszyński,^a^ Linfeng Hu,^f^ Rudolf Holze,^g,h^ Xuecheng Chen^a,c^**

^a^ Faculty of Chemical Technology and Engineering, West Pomeranian University of Technology, Szczecin, Piastów Ave. 42, 71-065 Szczecin, Poland

^b^ NanoBioMedical Centre, Adam Mickiewicz University, Wszechnicy Piastowskiej 3, 61-614 Poznan, Poland

^c^ Center for Advanced Materials and Manufacturing Process Engineering (CAMMPE), West Pomeranian University of Technology in Szczecin, Szczecin, Poland

^d^ Department of Science and Technology, Laboratory of Organic Electronics (LOE), Linköping University, 60174 Norrköping, Sweden

^e^ State Key Laboratory of Inorganic Synthesis and Preparative Chemistry, College of Chemistry, Jilin University, Changchun, 130012, Jilin Province, China

^f^ School of Materials Science and Engineering, Southeast University, Nanjing 211189, China

^g^ Chemnitz University of Technology, Chemnitz, D-09107 Germany

^h^ State Key Laboratory of Materials-oriented Chemical Engineering, School of Energy Science and Engineering, Nanjing Tech University, Nanjing, 211816, Jiangsu Province, China

**
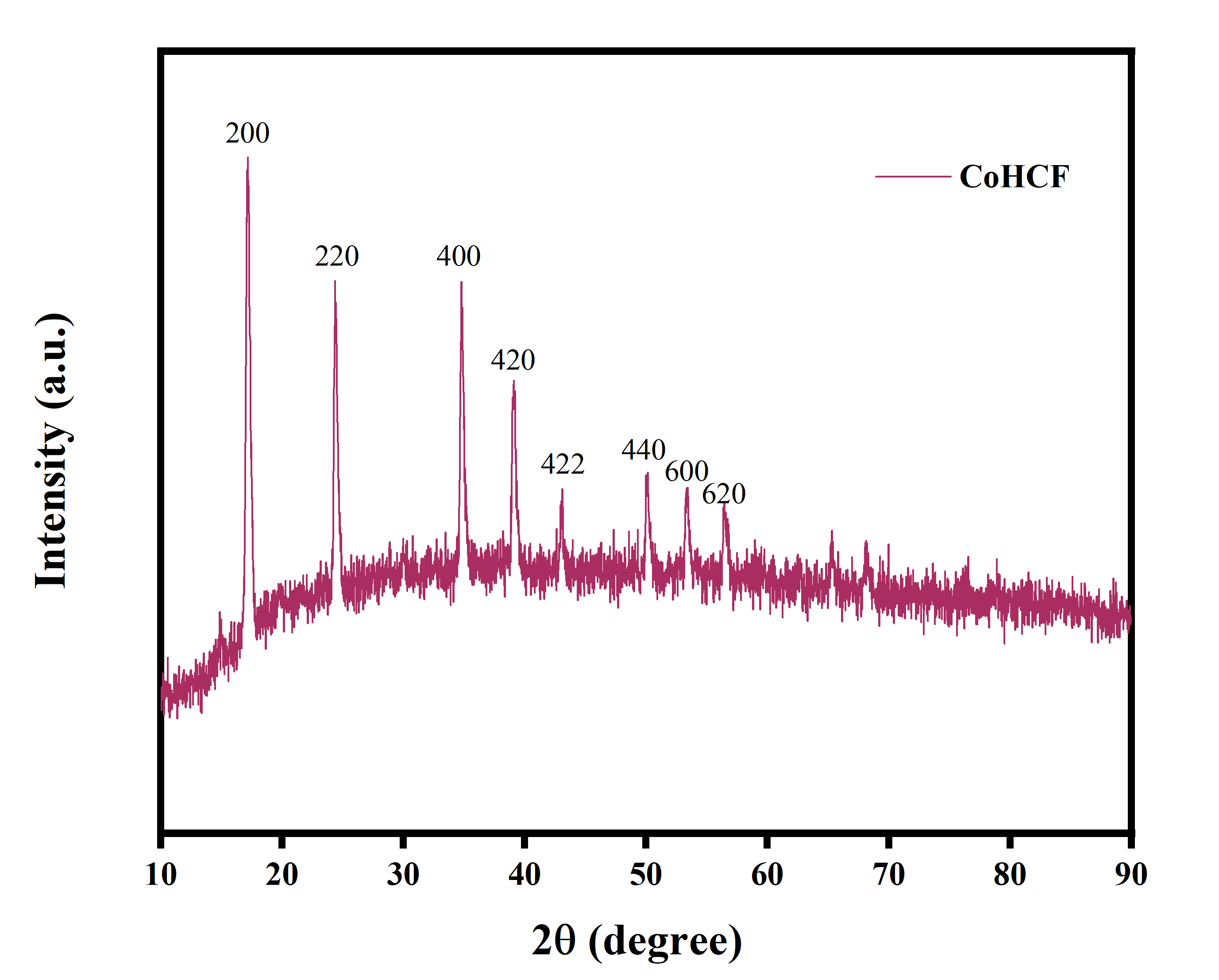
**

**Figure S1.** XRD pattern of Prussian blue analog (PBA) cathode materials.


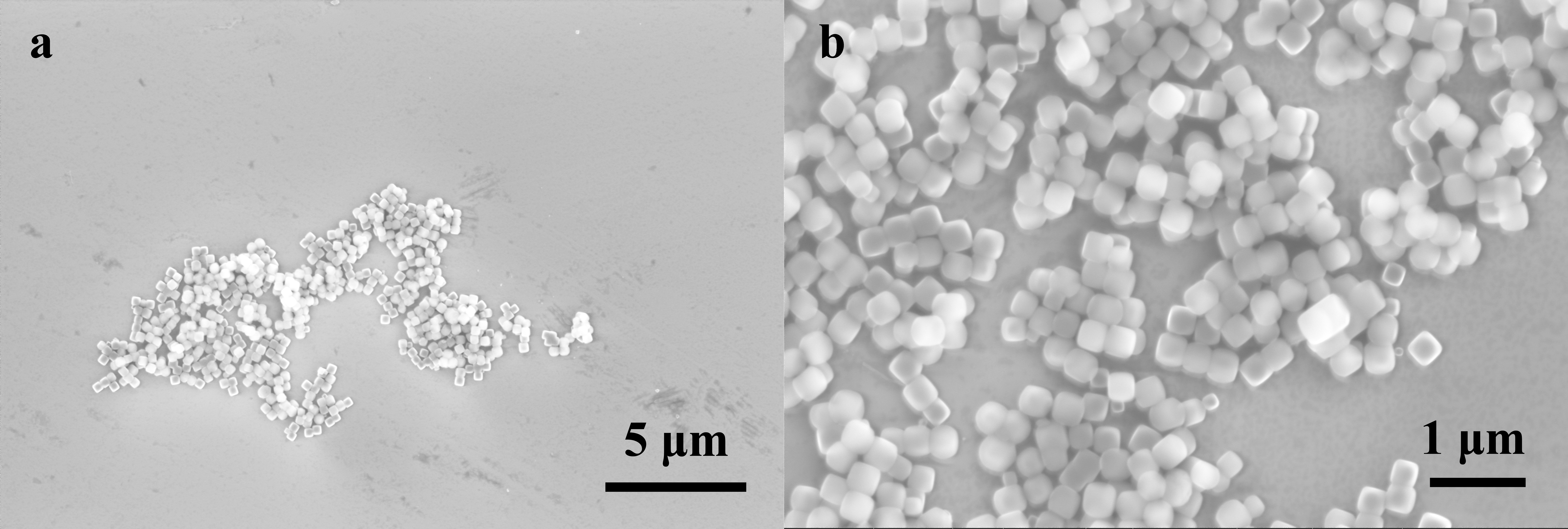


**Figure S2.** SEM micrographs of PBA cathode materials at different scales. a) 5 μm and b) 1 μm.


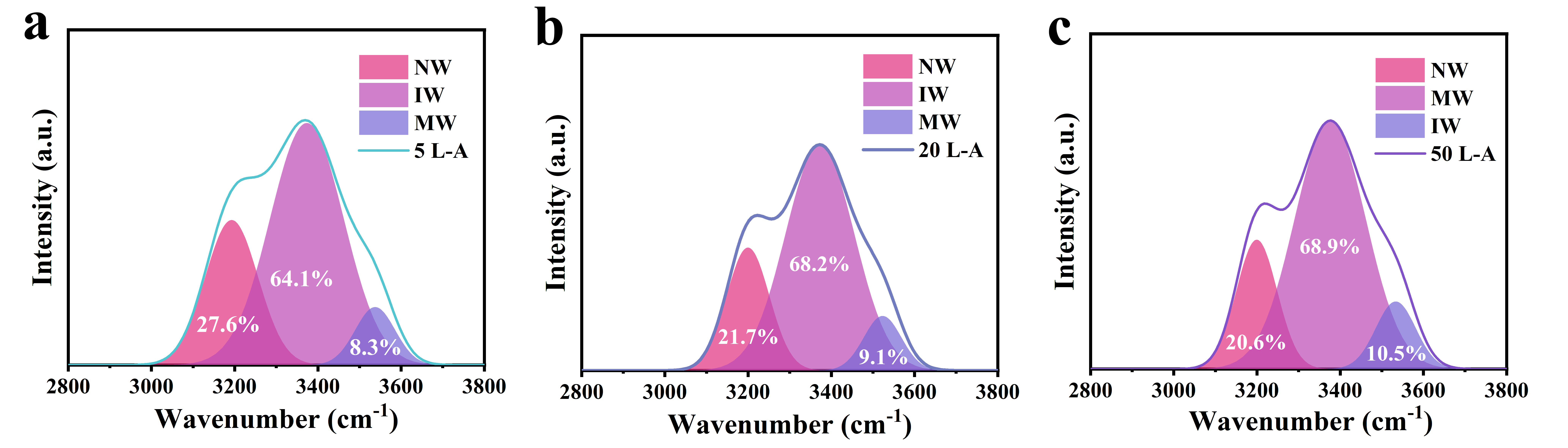


**Figure S3.** The fitted FT-IR spectra of water from electrolytes with different concentrations of additives. a) 5 L-A. b) 20 L-A and c) 50 L-A.


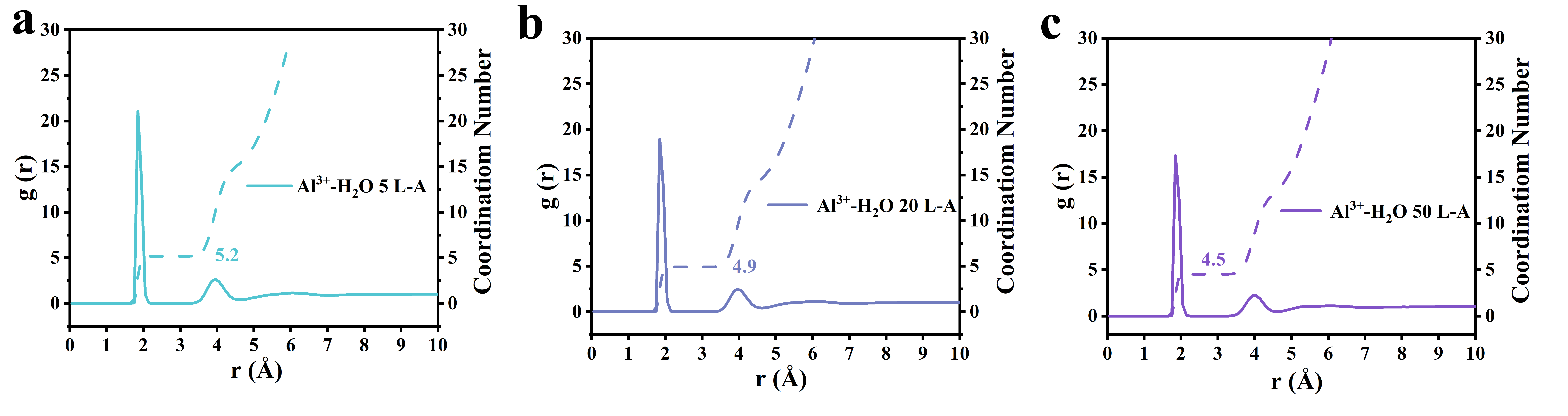


**Figure S4.** Radial distribution functions and coordination numbers of Al-H_2_O in 5 L-A, 20 L-A and 50 L-A electrolytes. a) 5 L-A. b) 20 L-A and c) 50 L-A.


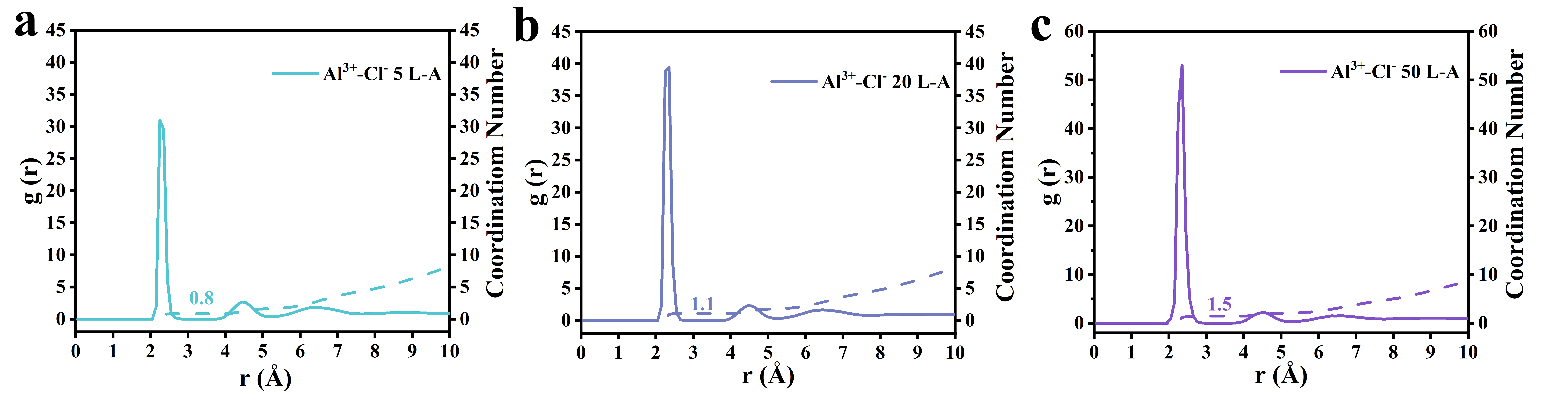


**Figure S5.** Radial distribution functions and coordination numbers of Al-Cl in 5 L-A, 20 L-A and 50 L-A electrolytes. a) 5 L-A. b) 20 L-A and c) 50 L-A.


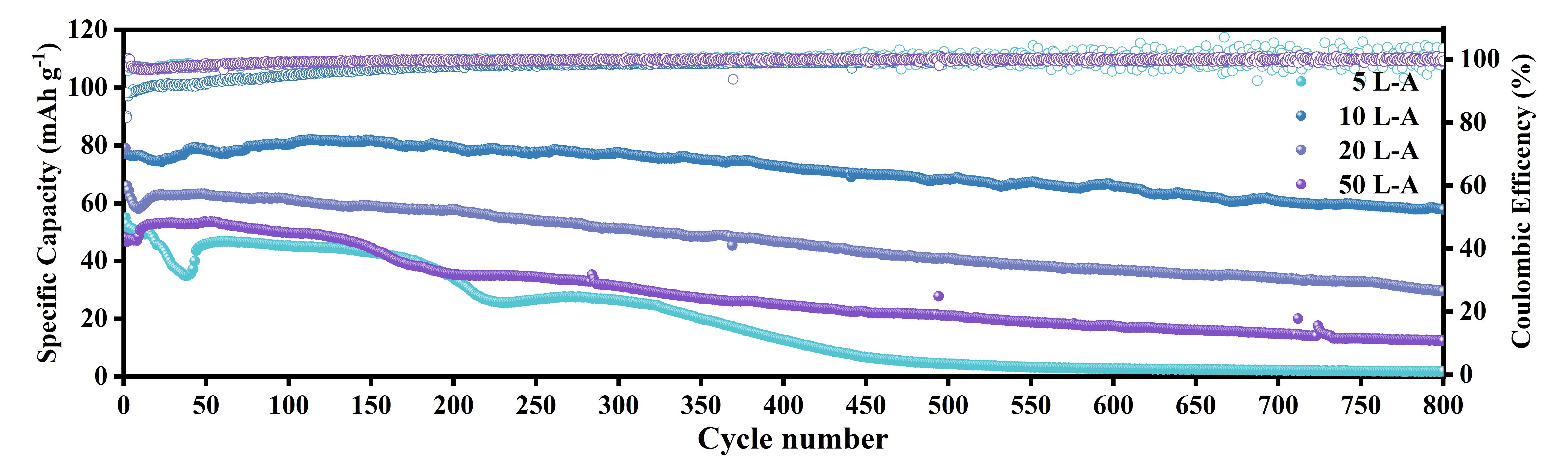


**Figure S6.** Cycling performance of aluminum-metal batteries assembled using hybrid electrolytes with different concentrations of additives at a current density of 250 mA g^-1^.


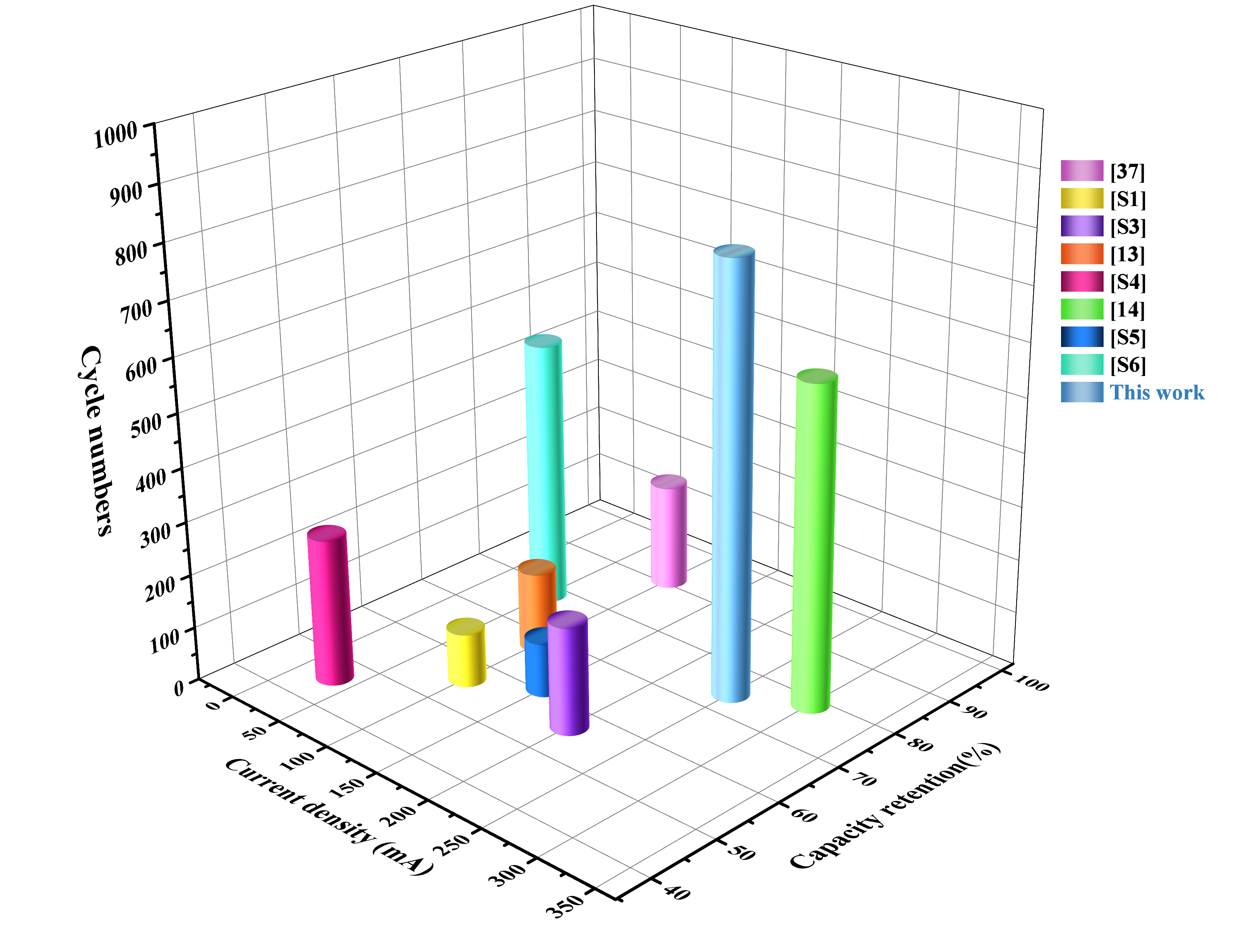


**Figure S7** 10 L-A full battery vs. electrochemical cycling performance of reported research.


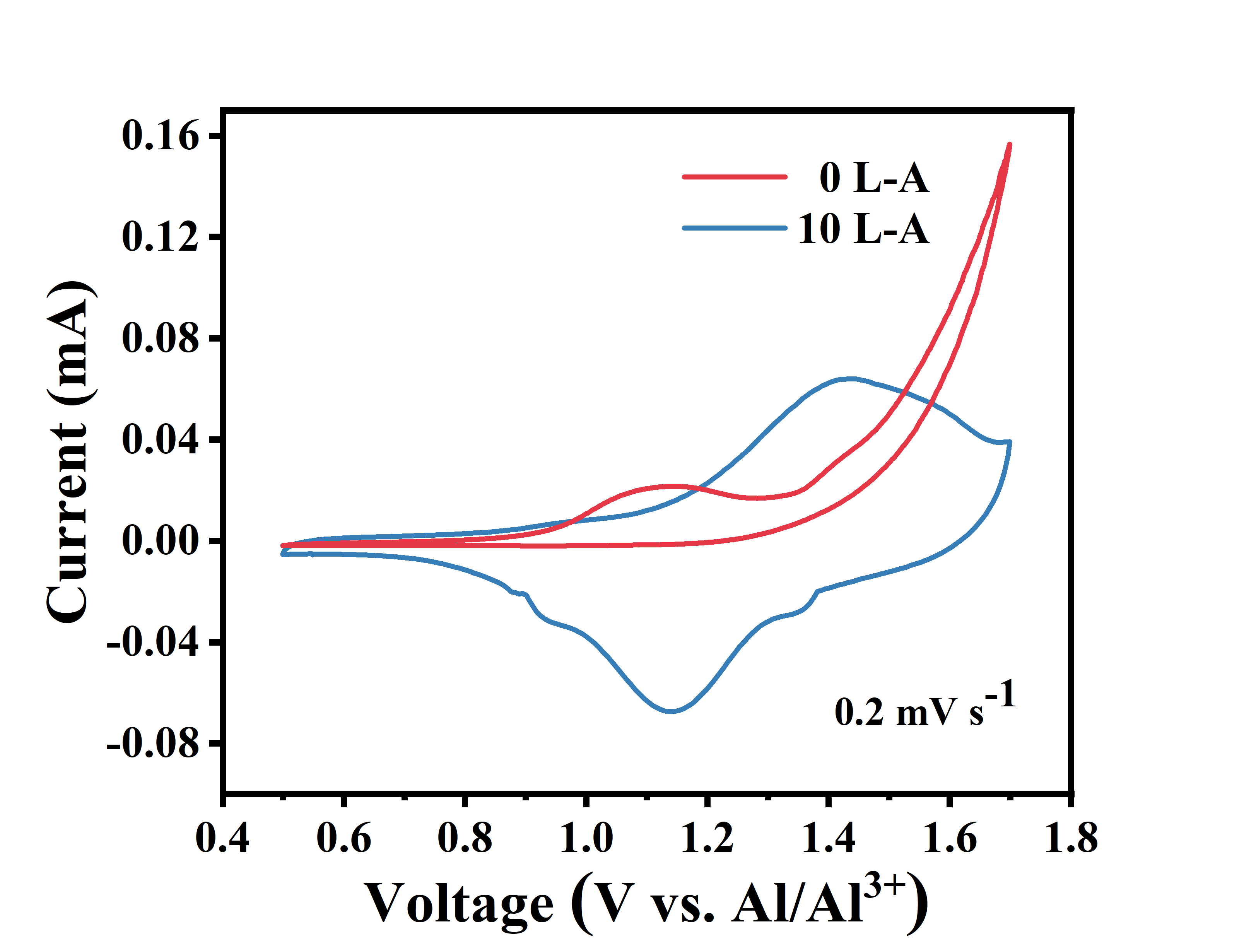


**Figure S8** Cyclic voltammetry curves for 0 L-A and 10 L-A at a scan rate of 0.2 mV s^−1^.


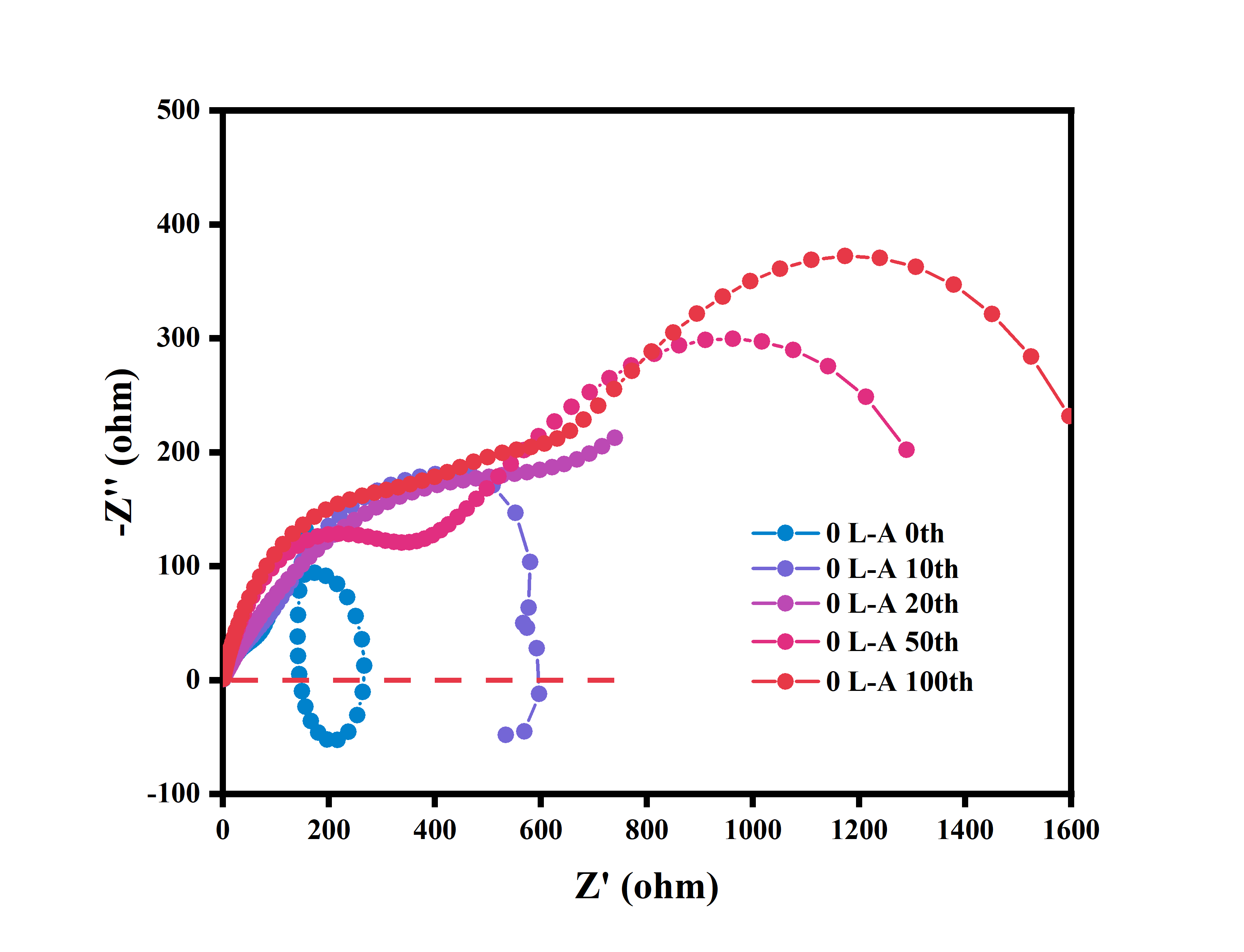


**Figure S9** Impedance spectrum: 0 L-A.


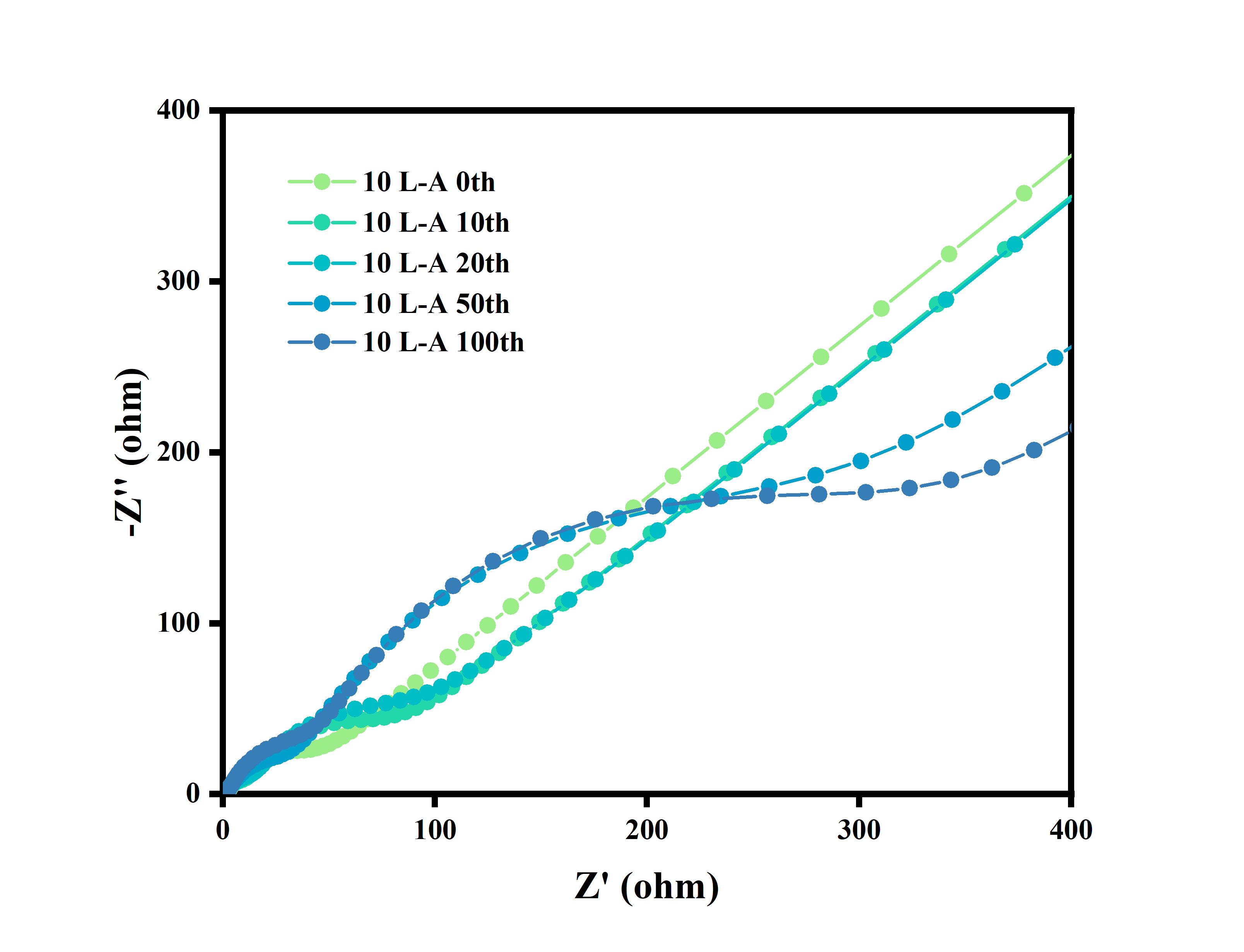


**Figure S10** Impedance spectrum: 10 L-A.


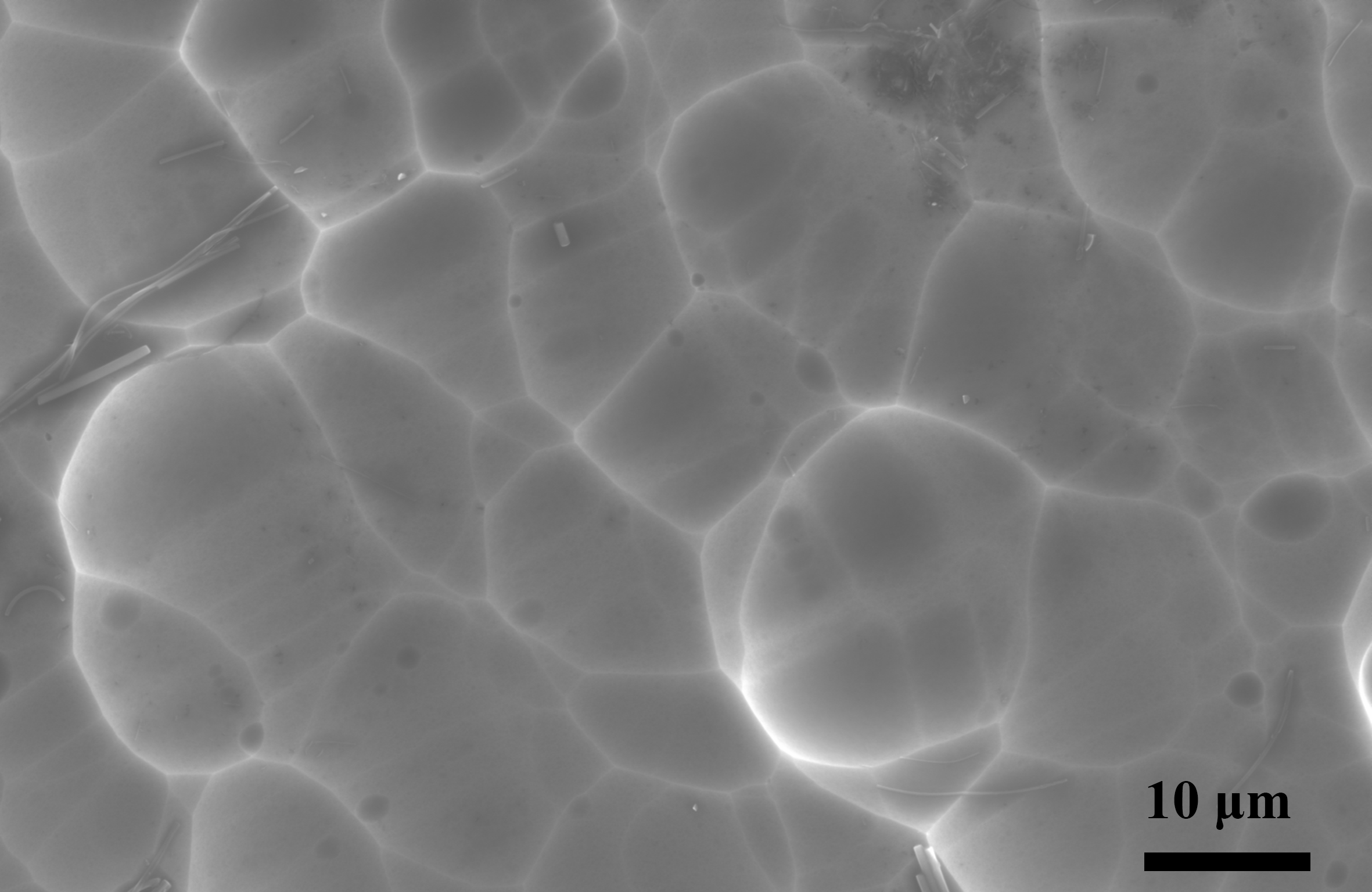


**Figure S11.** SEM image of the aluminum metal anode surface after 800th cycle of 10 L-A.


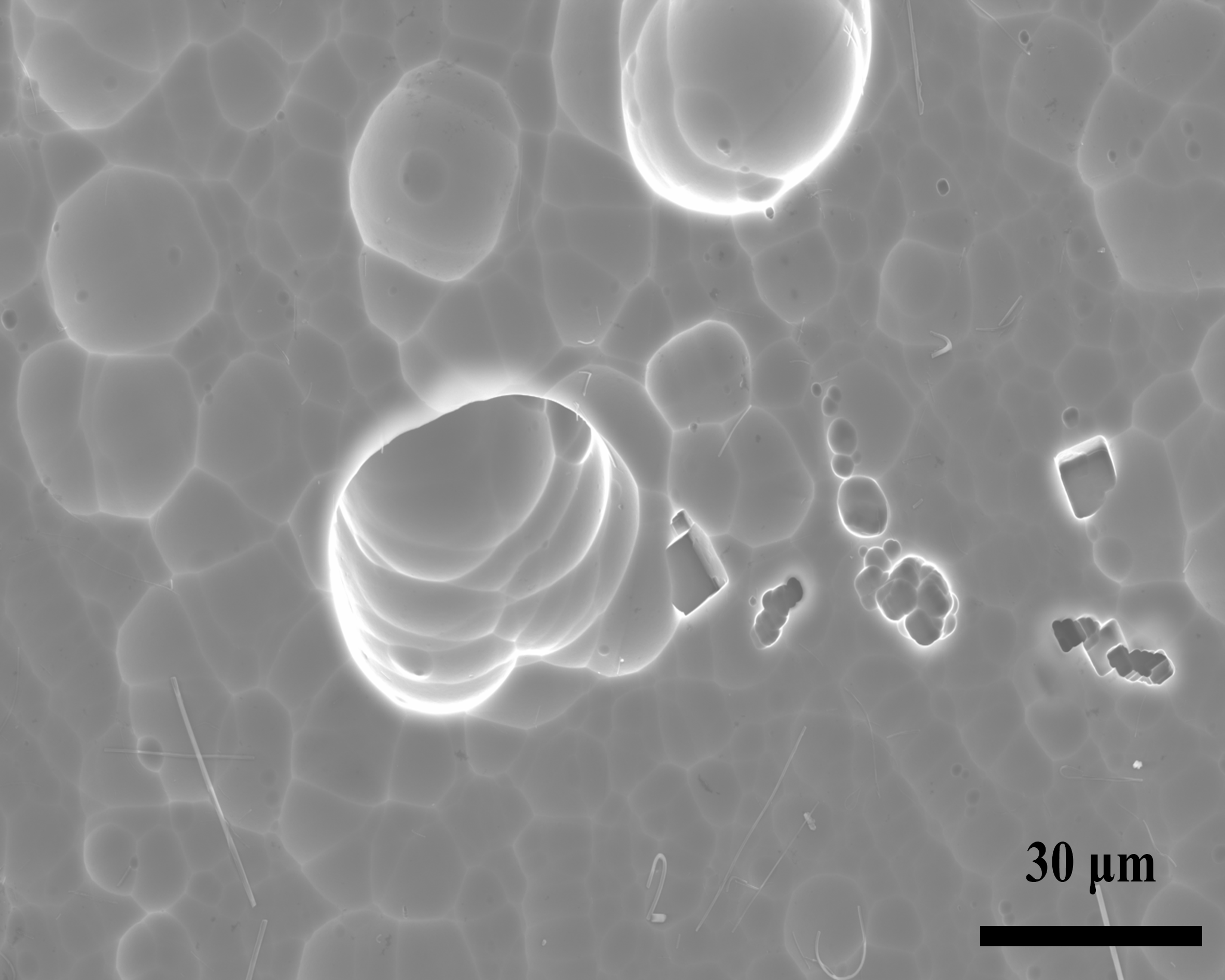


**Figure S12.** SEM image of localized surface pores of aluminum metal anode after 800th cycle of 10 L-A.


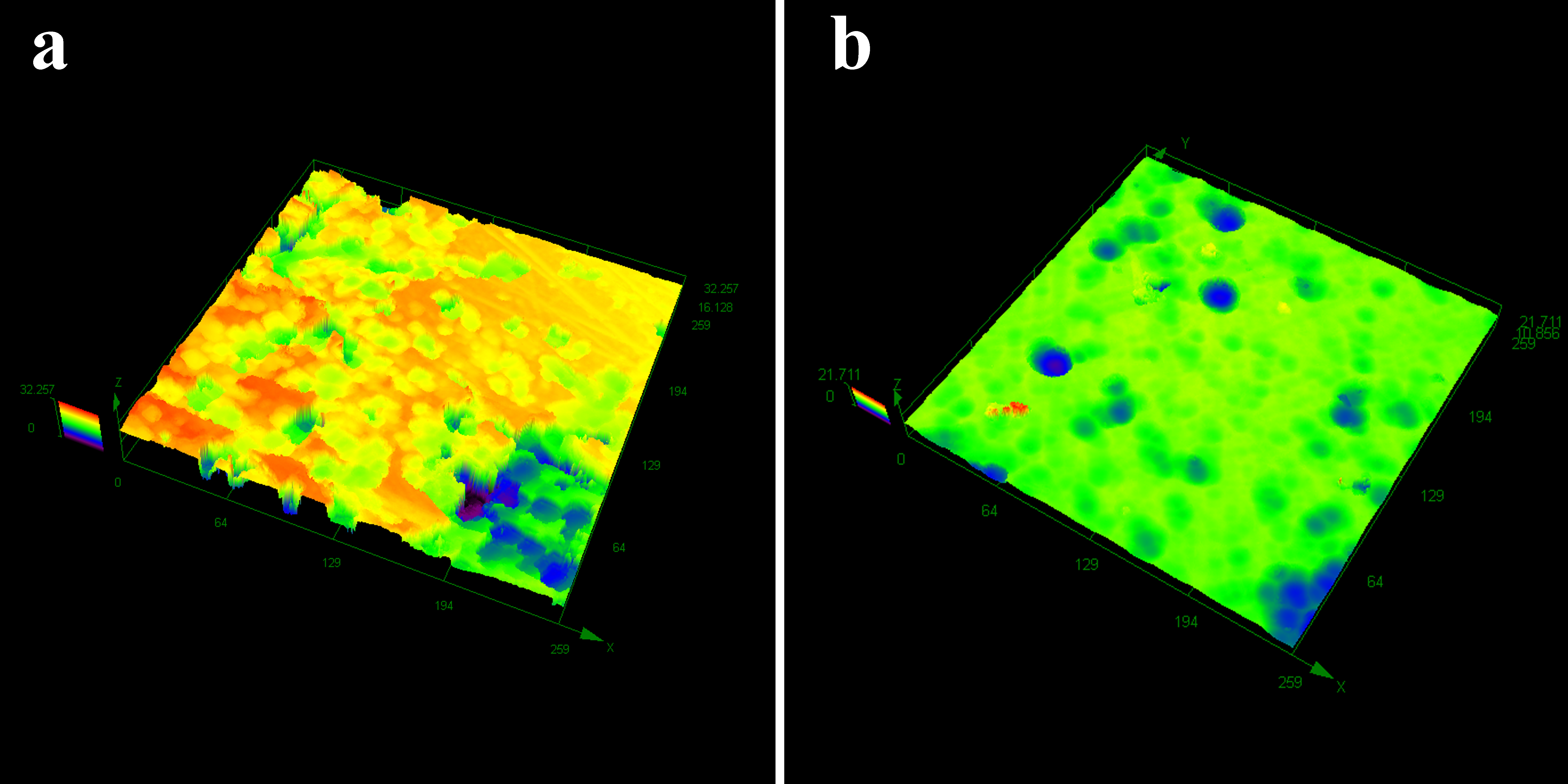


**Figure S13.** 3D CLSM images of aluminum metal anode electrodes with different electrolytes after 800 cycles. a): 0 L-A and b): 10 L-A.


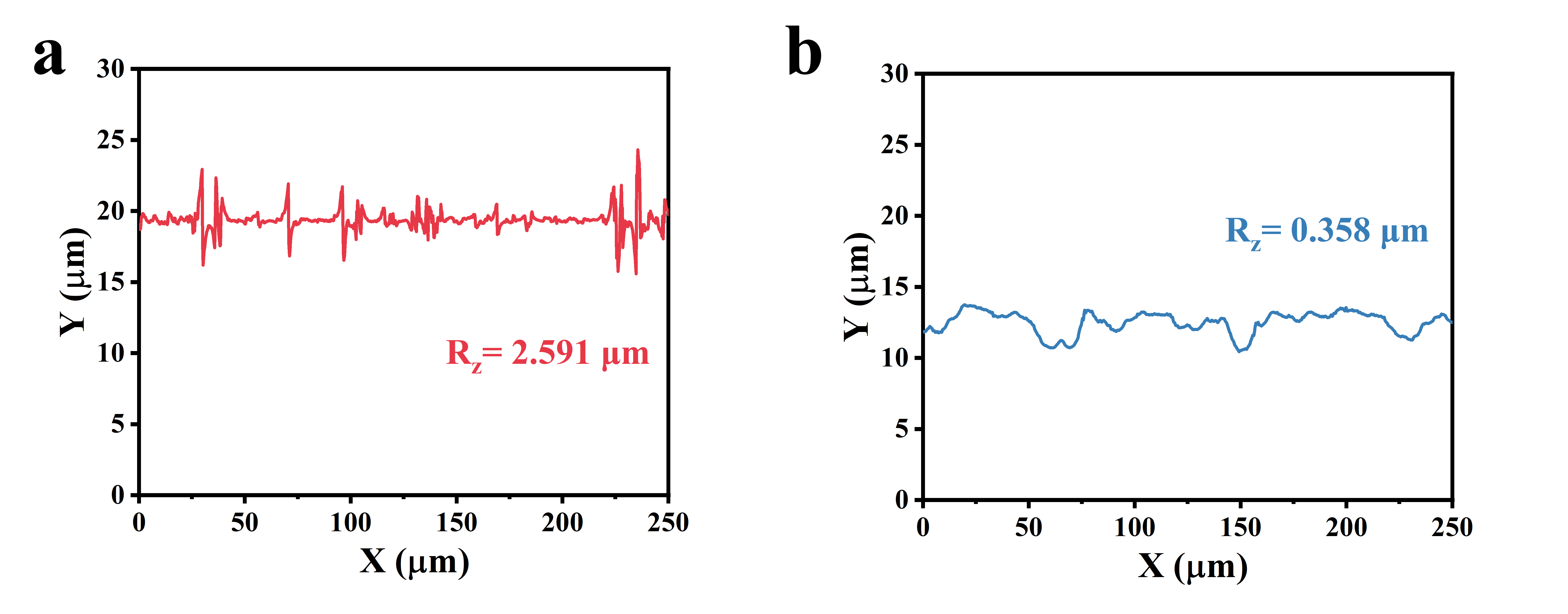


**Figure S14.** Roughness profiles of aluminum metal anode after 800 cycles in 0 L-A and 10 L-A electrolytes. a): 0 L-A and b): 10 L-A.


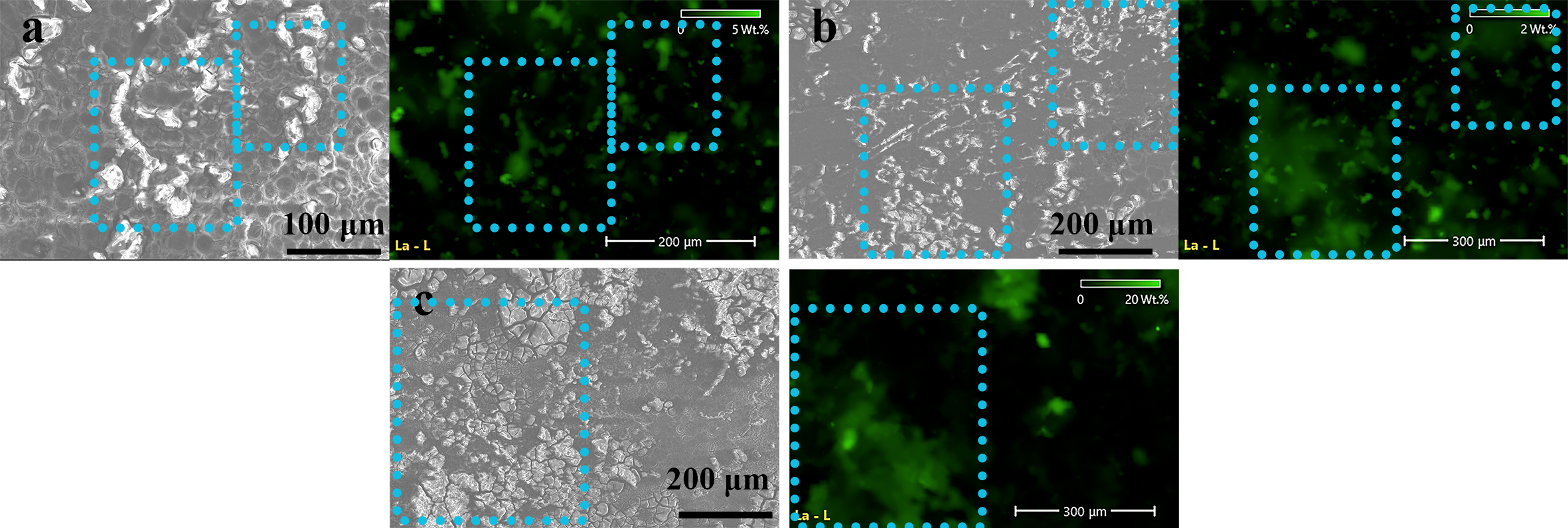


**Figure S15.** EDS spectra of the aluminum metal surface after 400 hours of operation at 0.1 mA cm^-2^ and 0.1 mAh cm^-2^ in symmetric batteries with different concentrations of LaCl_3_ additive, where the green color in the figures represents the La element. a) 10 L-A. b) 20 L-A and c) 50 L-A.


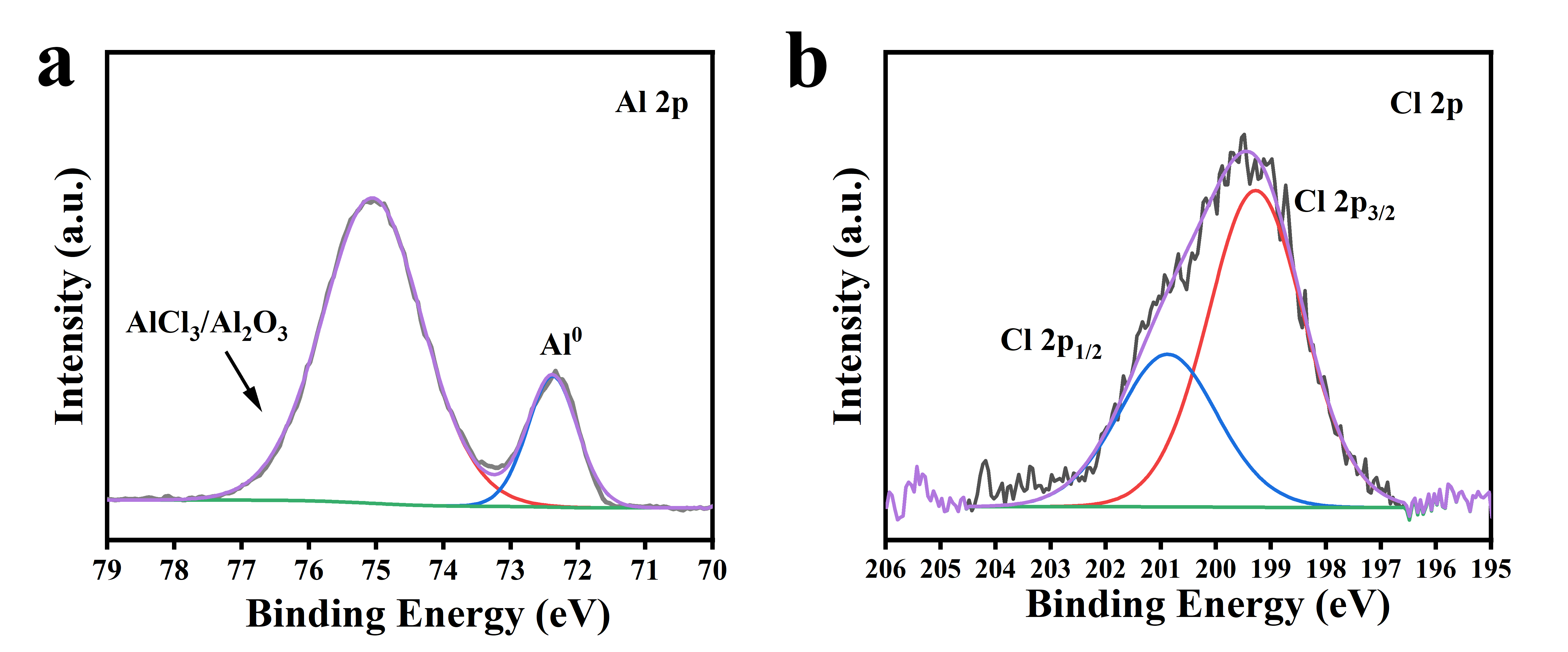


**Figure S16.** XPS spectra. a) Al 2p and b) Cl 2p.


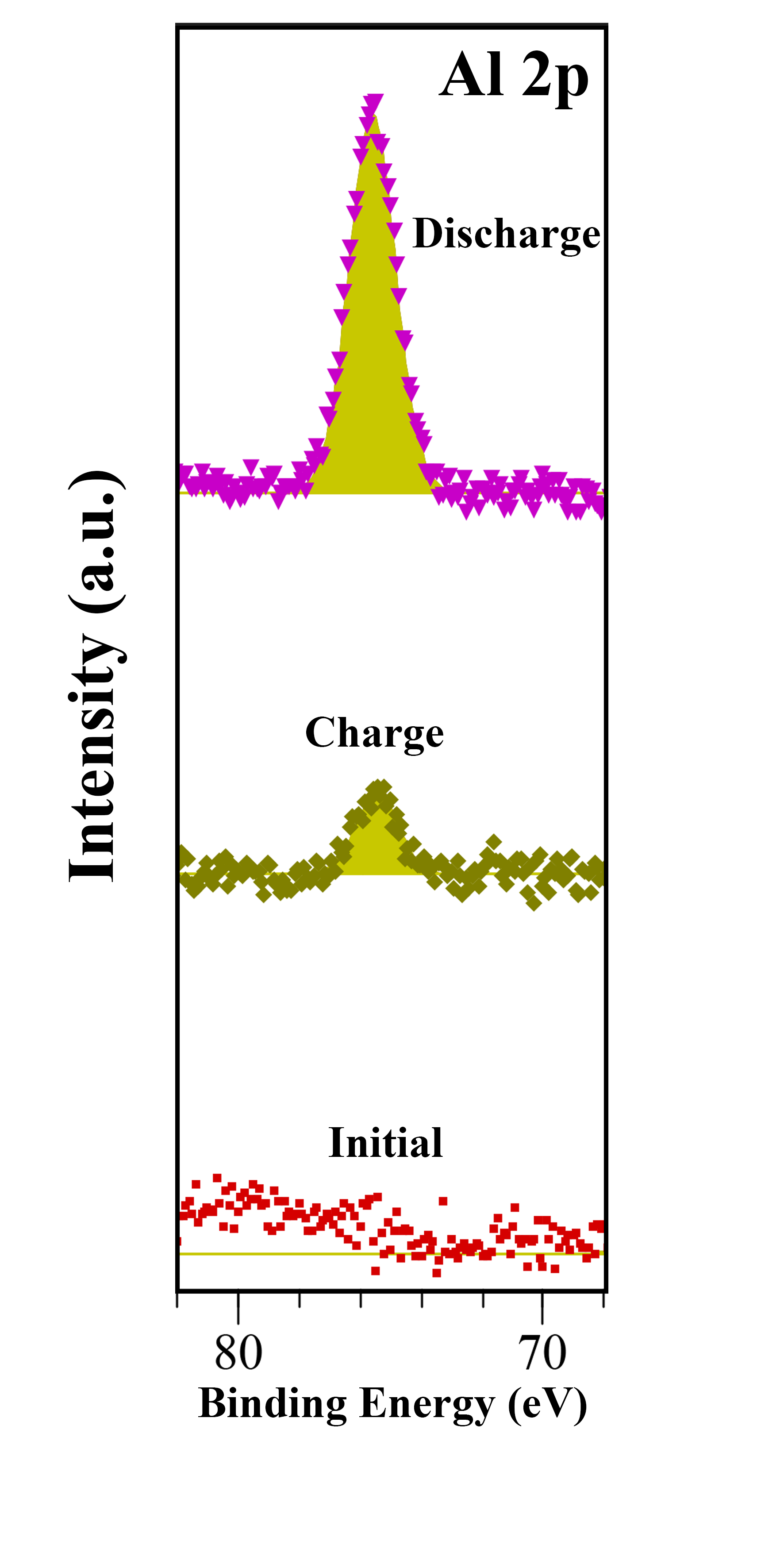


Figure S17. *Ex-situ* XPS analysis of Al 2p

Table S1 The performance of this work is compared with other aluminum metal batteries with PBA as cathode material

| Electrode materials | Electrolyte | Cut-off voltage | Current density | Capacity (mAh g^-1^)/cycle number | Capacity retention | ref |
| --- | --- | --- | --- | --- | --- | --- |
| KNiHCF/Amorphous Al@Al | 0.5 M Al_2_(SO_4_)_3_ | 1.4 V | 100 mA g^-1^ | 58/200/52.8 | 91.0% | 37 |
| FeHCF/Eutectic-treated Al | 2 M AlTFS | 0.3-1.9 V | 100 mA g^-1^ | 80/100/45 | 56.3% | S1 |
| KNHCF/Al | 5 M Al(OTF)_3_ | 0.2-1.1 V | 20 mA g^-1^ | 46.5/500/~25 | 53.8% | S2 |
| Defective MnFe-PBA/IL-treated Al | 1 M Al(OTF)_3_ hydrogel | 0.6-1.75 V | 200 mA g^-1^ | 64.7/200/36.5 | 56.4% | S3 |
| FeFeCN_6_/Treated Al | 2 M Al(OTF)_3_ | 0.2-1.5 V | 100 mA g^-1^ | 85/150/57.8 | 68% | 13 |
| NMHCF/IL Treated Al | 2 M Al(OTF)_3_ | 0.6-1.9 V | 30 mA g^-1^ | 72/275/33.1 | 46% | S4 |
| CuHCF/Al | 2 M Al(OTF)_3_+20 M LiTFSI | 0.1-1.8 V | 200 mA g^-1^ | 94/75/＜50 | ＜53.2% | 19 |
| CuHCF/Treated Al | 1 M Al(OTF)_3_ | 0.6-1.5 V | 300 mA g^-1^ | -/600/- | 78.7% | 14 |
| FF-PBA/Al | Water in salt electrolyte | Three-electrode system | 150 mA g^-1^ | 116.29/100/70.72 | 60.8% | S5 |
| CoFeCN_6_/Al | TEP-Al(OTF)_3_ | 0.1-1.3 V | 50 mA g^-1^ | 66.6/500/51.9 | 78% | S6 |
| KNHCF/Al | Al(OTF)_3_ hydrogel electrolyte | ~0.2~1.5V | 100 mA g^-1^ | 74.9/200/ | 90% | S7 |
| CoHCF/Al | 10 mM LaCl_3_+1 M AlCl_3_ | 0.5-1.7 V | 250 mA g^-1^ | 77.7/500/68.3  77.7/800/57.8 | 87.9%  74.4% | This work |

**References**

S1. S. Kumar, T. Salim, V. Verma, W. Manalastas and M. Srinivasan, Enabling Al-metal Anodes for Aqueous Electrochemical Cells by Using Low-Cost Eutectic Mixtures as Artificial Protective Interphase, Chemical Engineering Journal, 2022, 435, <https://doi.org/10.1016/j.cej.2022.134742>

S2. Y. Gao, H. Yang, X. Wang, Y. Bai, N. Zhu, S. Guo, L. Suo, H. Li, H. Xu and C. Wu, The Compensation Effect Mechanism of Fe-Ni Mixed Prussian Blue Analogues in Aqueous Rechargeable Aluminum-Ion Batteries, ChemSusChem, 2020, 13, 732, <https://doi.org/10.1002/cssc.201903067>

S3. D. Wang, H. Lv, T. Hussain, Q. Yang, G. Liang, Y. Zhao, L. Ma, Q. Li, H. Li, B. Dong, T. Kaewmaraya and C. Zhi, A Manganese Hexacyanoferrate Framework with Enlarged Ion Tunnels and Two‐Species Redox Reaction for Aqueous Al-Ion Batteries, Nano Energy, 2021, 84, <https://doi.org/10.1016/j.nanoen.2021.105945>

S4. S. Kumar, V. Verma, H. Arora, W. Manalastas and M. Srinivasan, Rechargeable Al-Metal Aqueous Battery Using NaMnHCF as a Cathode: Investigating the Role of Coated-Al Anode Treatments for Superior Battery Cycling Performance, ACS Applied Energy Materials, 2020, 3, 8627, <https://doi.org/10.1021/acsaem.0c01240>

S5. A. Zhou, L. Jiang, J. Yue, Y. Tong, Q. Zhang, Z. Lin, B. Liu, C. Wu, L. Suo, Y. S. Hu, H. Li and L. Chen, Water-in-Salt Electrolyte Promotes High-Capacity FeFe(CN)_6_ Cathode for Aqueous Al-Ion Battery, ACS Appl Mater Interfaces, 2019, 11, 41356, <https://doi.org/10.1021/acsami.9b14149>

S6. X. Xu, Q. Pang, X. Liu, P. Ma, X. Jiang, F. Xin, H. Wang, M. Xing, Y. Fu and Y. Tian, A Long-Lifespan Prussian Blue-Based Aluminum Metal Battery using an Aqueous/Organic Hybrid Electrolyte, Electrochimica Acta, 2025, 512, <https://doi.org/10.1016/j.electacta.2024.145503>

S7. Z. Wen, F. Wu, M. F. Ng, B. Jia, J. Song, T. Yu, J. Dong, A. Tang, R. Chen and Q. Yan, Lean-Water Hydrogel with Multipolar Sites for Flexible and High-Performance Aqueous Aluminum Ion Batteries, Adv Mater, 2025, 37, 2500695, <https://doi.org/10.1002/adma.202500695>
